# Local Exosome Inhibition Potentiates Mild Photothermal Immunotherapy Against Breast Cancer

**DOI:** 10.1002/advs.202406328

**Published:** 2024-11-22

**Authors:** Qian Chen, Yanan Li, Jiameng Hu, Zhenyu Xu, Shengyi Wang, Naicong Cai, Mengjiao He, Yifang Xiao, Yuan Ding, Mengjuan Sun, Chunjiayu Li, Yiyang Cao, Zhongyuan Wang, Fang Zhou, Guangji Wang, Chen Wang, Jiasheng Tu, Haiyang Hu, Chunmeng Sun

**Affiliations:** ^1^ State Key Laboratory of Natural Medicines China Pharmaceutical University 639 Longmian Avenue Nanjing 211198 China; ^2^ Department of Pharmaceutics School of Pharmacy China Pharmaceutical University 639 Longmian Avenue Nanjing 211198 China; ^3^ NMPA Key Laboratory for Research and Evaluation of Pharmaceutical Preparations and Excipients China Pharmaceutical University 24 Tong Jia Xiang Nanjing 210009 China; ^4^ Jiangsu Provincial Key Laboratory of Drug Metabolism and Pharmacokinetics China Pharmaceutical University 24 Tong Jia Xiang Nanjing 210009 China; ^5^ School of Food and Pharmaceutical Engineering Nanjing Normal University No. 1 Wenyuan Road Nanjing 210023 China; ^6^ School of Life Science and Technology China Pharmaceutical University 639 Longmian Avenue Nanjing 211198 China; ^7^ Institute of Systems and Physical Biology Shenzhen Bay Laboratory Shenzhen 518055 China; ^8^ Department of Pharmaceutics School of Pharmacy Fudan University Shanghai 201203 China; ^9^ Central Laboratory Shanghai Pulmonary Hospital Tongji University School of Medicine Shanghai 200433 China

**Keywords:** combination immunotherapy, exosome inhibition, immune checkpoint blockade, mild photothermal therapy, multistage drug depot

## Abstract

Limited immune infiltration within the tumor microenvironment (TME) hampers the efficacy of immune checkpoint blockade (ICB) therapy. To enhance immune infiltration, mild photothermal therapy (PTT) is often combined with immunotherapy. However, the impact of mild PTT on the TME remains unclear. The bioinformatics analyses reveal that mild PTT amplifies immune cell infiltration and stimulates T‐cell activity. Notably, it accelerates the release of tumor cell‐derived exosomes (T_EX_) and upregulates PD‐L1 expression on both tumor cells and T_EX_. Consequently, it is proposed that locally inhibiting T_EX_ release is crucial for overcoming the adverse effects of mild PTT, thereby enhancing ICB therapy. Thus, a multi‐stage drug delivery system is designed that concurrently delivers photosensitizers (reduced graphene oxide nanosheets, NRGO), anti‐PD‐L1 antibodies, and exosome inhibitors (sulfisoxazole). The system employs a temperature‐sensitive lipid gel as the primary carrier, with NRGO serving as a secondary carrier that supports photothermal conversion and incorporation of sulfisoxazole. Importantly, controlled drug release is achieved using near‐infrared radiation. The findings indicate that this local combination therapy remodels the immunosuppressive TME through exosome inhibition and enhanced immune cell infiltration, while also boosting T‐cell activity to trigger systemic antitumor immunity, showcasing the remarkable efficacy of this combination strategy in eradicating cold tumors.

## Graphical abstract

Exosome inhibition is required to potentiate mild photothermal‐based ICB immunotherapy. Herein, a multi‐stage drug delivery system is designed to concurrently deliver photosensitizers (reduced graphene oxide nanosheets, NRGO), anti‐PD‐L1 antibodies, and exosome inhibitors (sulfisoxazole) in an all‐in‐one and all‐in‐control manner.

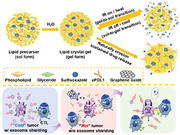

## 1 Introduction

Tumor immunotherapy works by stimulating the host's immune system to generate a systemic immune response against the spread of tumor cells.^[^ ^1^ ^]^ Among various immunotherapies, ICB therapy based on the PD‐1/PD‐L1 pathway has made important clinical advances. However, limited by a highly immunosuppressive TME and low immune cell infiltration, only a small percentage of patients benefit from a single ICB therapy.^[^ ^2^ ^]^ Numerous studies have found a high correlation between ICB efficacy and the proportion of T cell infiltration in tumor tissues,^[^ ^3^ ^]^ thus how to effectively increase T cell infiltration and stimulate T cell activation is of great importance for ICB therapy in solid tumors.

Mild PTT is an emerging therapeutic modality that uses a temperature slightly higher than body temperature (≈45 °C) to heat tumor tissues for therapeutic effects.^[^ ^4^ ^]^ Unlike the direct ablative effect of high temperature, mild PTT aims to increase immune cell infiltration and activate anti‐tumor immune response by increasing blood perfusion and improving vascular permeability.^[^ ^5^ ^]^ Therefore, mild PTT is often used to increase the infiltration of T cells and CAR‐T cells in solid tumors.^[^ ^5^, ^6^ ^]^ In our previous study, we designed an all‐in‐one and all‐in‐control strategy to alleviate the immunosuppression of TME in situ by the effect of mild PTT on tumors, which turned cold tumors into hot and enhanced immunotherapy.^[^ ^4a^ ^]^

However, the mechanisms of mild PTT for modulating immune cells in TME remain unclear. It is also unknown whether mild PTT also leads to any negative therapeutic effect during the treatment process. To maximize the synergistic effect of mild PTT‐potentiated combination immunotherapy, an in‐depth investigation of the influence of mild PTT on TME, including the biological function changes of tumors and immune cells, is imperative.

The exosome is a class of extracellular vesicles with particle size of 40–160 nm.^[^ ^7^ ^]^ It has been reported that external stimuli, i.e., pH, temperature, and hypoxia, often accelerate the release of exosomes.^[^ ^8^ ^]^ Tumor cell‐derived exosomes (T_EX_) play important roles in immune escape for the high expression of PD‐L1 on the surface and various miRNA contents, which could mediate immune evasion and regulate immune cell phenotypes.^[^ ^9^ ^]^ Thus, T_EX_ was supposed to negatively modulate the immune effect of mild PTT.

Therefore, in this study, we first investigated the immune modulation effect of in situ mild PTT on immune cells in TME by single‐cell RNA sequencing (scRNA‐seq) and bulk RNA sequencing (bulk RNA‐seq). Mild PTT significantly influences the intricate immune systems, leading to the activation of dendritic cells (DCs) and T cells. This observation suggests that immunotherapy could benefit from mild PTT. To strike a balance between the benefits and drawbacks of mild PTT, we introduced synchronized exosome inhibition to maximize its effect. To verify the above hypothesis, we developed an all‐in‐one and all‐in‐control strategy by combining mild PTT, exosome inhibition, and ICB therapy within a rational lipid gel depot. Specifically, we utilized an injectable temperature‐sensitive LG developed in a previous study as an in situ drug reservoir for encapsulating the anti‐PD‐L1 antibody (aPDL1), photosensitizer (reduced graphene oxide nanosheets, NRGO),^[^ ^10^ ^]^ and exosome inhibitor (sulfisoxazole, SFX).^[^ ^11^ ^]^ We first prepared precursor gel formulations by mixing the above drugs with lipids and administered them in an all‐in‐one modality, where the precursor agents rapidly formed gels when they came in contact with water or tissue fluid.

**Figure 1:**
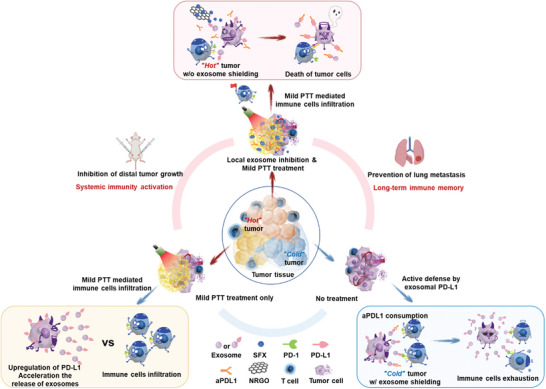
Schematic illustration of combination immunotherapy based on reshaping of tumor microenvironment by mild photothermal therapy along with exosome inhibition to potentiate immune checkpoint blockade therapy.

Among them, NRGO could serve multiple roles. Due to their unique 2D structure, they can act as secondary drug carriers for loading SFX. Moreover, NRGO also interacts with the immune system. Notably, it acts as a nano‐adjuvant, robustly stimulating cellular immunity.^[^ ^12^ ^]^ Furthermore, NRGO significantly promotes the secretion of both Th1 and Th2 cytokines and chemokines, thereby enhancing both adaptive and innate immune responses.^[^ ^13^ ^]^ Additionally, NRGO possesses excellent photothermal conversion ability, enabling mild PTT with immunomodulatory function.^[^ ^14^ ^]^ The temperature‐sensitive LG allows mild PTT of NRGO to regulate the drug release from the in situ reservoir, functioning as a drug release switch. As a result, mild PTT serves as a bridge between immune function and drug release behavior, creating a flexible all‐in‐control modality. Therefore, we developed an exosome inhibition strategy to synergistically neutralize the side effects of thermally triggered release of T_EX_, alleviate the immunosuppression of TME, and improve the efficacy of ICB therapy (**Figure** **1**). We validated this synergistic immunotherapy strategy in tumor‐bearing mouse models of primary, distal, and metastatic breast cancer. The results demonstrated that combining mild PTT with exosome inhibition is an effective approach to enhance the efficacy of ICB therapy.

## 2 Results

### 2.1 Immune Landscape in TME After Mild PTT

**Figure 2:**
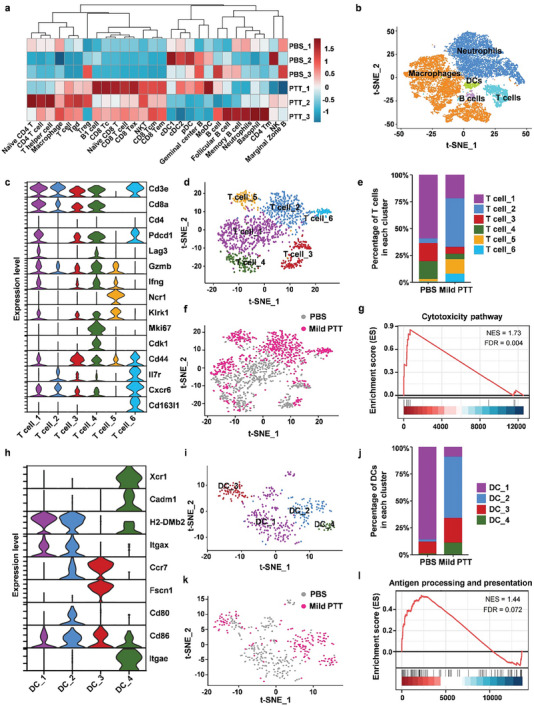
Bulk RNA‐seq and scRNA‐seq analysis of immune cells in TME after the treatment of mild PTT. a) Heat map of the immune cells in TME analyzed by bulk RNA‐seq. b) the t‐SNE plot of the main immune cell types from all samples. Each cluster is colored and annotated according to cell type. c) Violin plot of representative marker genes of each cluster of T cells. d) the t‐SNE plot of T cells obtained from the PBS group and mild PTT group. Phenotypic clusters are represented in distinct colors. e) Bar plot of the frequency of T cells within each cluster in the PBS group and Mild PTT group. f) Clustering of T cells derived from PBS group and mild PTT group, and t‐SNE plot colored by sample origins. g) GSEA analysis of T cells and enrichment plots for cytotoxicity pathway. h) Violin plot of representative marker genes of each cluster of DCs. i) t‐SNE plot of DCs obtained from PBS group and Mild PTT group. Phenotypic clusters are represented in distinct colors. j) Bar plot of the frequency of DCs within each cluster in the PBS group and mild PTT group. k) Clustering of DCs derived from PBS group and mild PTT group, and t‐SNE plot colored by sample origins. l) GSEA analysis of DCs and enrichment plots for antigen processing and presentation.

To elucidate the influence of mild PTT effect on the immune landscape in TME, we applied bulk RNA‐seq to decipher immune cell infiltration in tumor tissues based on the proportion of RNA sequences belonging to unique cell populations. The heatmap of immune cell abundance in each sample is shown in **Figure** **2**a. Among them, the levels of T cells were higher in the mild PTT group in comparison with the PBS group, indicating that mild PTT could significantly promote the recruitment and activation of T cell subtypes. These studies suggest that T cells are the important subsets of immune cells participating in mild PTT‐potentiated immune responses.

To investigate more specific details of immune cells in TME, scRNA‐seq was carried out to characterize cell type‐specific transcriptional profiles of the PBS group and mild PTT group. The dimensionality reduction technique t‐distribution stochastic neighbor embedding (t‐SNE) was applied to characterize the single cells and illustrate genome‐wide similarities and differences in their gene expression profiles. As depicted in Figure 2b, we can identify five major cell types including T cells, macrophages, DCs, neutrophils, and B cells.

T cells play important roles in tumor‐specific immune response, we first focused on the subtypes of T cells. In‐depth scRNA‐seq clustering of the T cell population yielded six subclusters (Figure 2c–f). T cells in the group of mild PTT were mainly located in T cell_2, which expressed cytotoxicity marker *Gzmb* and without the expression of exhausted markers *Pdcd1* and *Lag3*. T cell_5 and T cell_6 were also largely expanded by mild PTT effect, of which T cell_5 presented an NKT phenotype with high expression of *Ncr1* and *Klrk1*, T cell_6 presented a γδT phenotype with high expression of *Cxcr6* and *Cd163l1*. The proportions of T cell_1, T cell_3, and T cell_4 were decreased after the exposure of mild PTT. T cell_1 and T cell_4 displayed high co‐expression of *Pdcd1* and *Lag3*, indicating an exhausted phenotype. Most T cells in the PBS group were located in T cell_1, which displayed a highly exhausted state. Furthermore, we further conducted Gene Set Enrichment Analysis (GSEA) to understand the biological characteristics discrepancy of T cell subsets in the PBS group and mild PTT group (Figure 2g). Excitingly, genes associated with the cytotoxicity pathway were enriched in the mild PTT group, indicating the enhancement of cytotoxicity. Thus, mild PTT could reshape the TME by inducing the infiltration of naïve T cells and improving the cytotoxicity of T cells.

DCs are the main regulators for initiating antigen‐specific immune responses in tumor immunity 19. We observed four clusters of DCs (Figure 2h–k). DCs from the mild PTT group are mainly located in DC_2, with the highest expression of co‐stimulatory markers *Cd80* and *Cd86* as well as the up‐regulated migration marker *Ccr7*, indicating the activation of DCs after the mild PTT treatment. Whereas, DCs from the PBS group are mainly located in DC_1, with the lowest expression of *Cd86* and seldom expression of *Cd80*, indicating the lowest degree of maturation. The ratios of DC_3 and DC_4 were both increased in the mild PTT group. DCs located in DC_3 with the high expression of *Ccr7* and *Fscn1* displayed a mregDC (mature DC enriched in regulatory molecules) signature, which was crucial for inducing tumor‐directed T‐cell responses. DCs located in DC_4, which highly expressed *Xcr1* and *Cadm1*, were characterized as cDC1, which was reported to present antigens mainly to CD8^+^ T cells. Indeed, our scRNA‐seq data also showed that DCs in tumors from the mild PTT group expressed an antigen‐presenting signature and related genes at a much higher level (Figure 2l).

Taken together, these data demonstrated that mild PTT therapy promoted antigen processing and presentation function of DCs, and expanded the populations of effector CD8^+^ T cells in TME.

### 2.2 Preparation and Characterization of the Multistage Drug Depot Based on Reduced Graphene Oxide Nanosheets and Thermal‐Responsive LG

**Figure 3:**
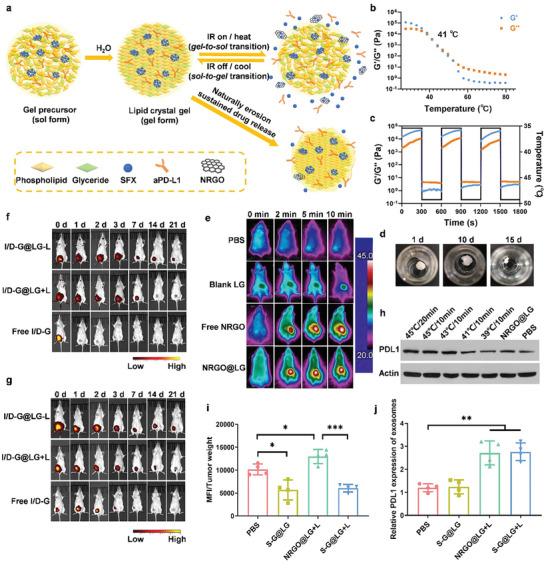
Preparation, characterization, photothermal performance, and drug release behavior of LG. a) Schematical illustration of the preparation and photothermal performance and drug release behavior of the LG. b) Temperature‐responsive storage (G′) and loss (G″) modulus changes of the LG with a PC/GDO mass ratio of 41/59. The phase transition temperature of the LG is 41 °C. c) Modulus changes of the LG with a PC/GDO mass ratio of 41/59 under three cycles of heating (45 °C) and cooling (37 °C). d) Morphology changes of the gel in PBS with 2% lipase over 15 days. e) In vivo infrared thermal images of the tumor sites in 4T1‐bearing mice irradiated after intratumor injection with PBS, blank LG, free NRGO, NRGO@LG. Images were recorded at 0 min, 2 min, 5 min, and 10 min after irradiation. f) IVIS images describing the intratumor retention time of IgG‐Cy3 in free I/D‐G and I/D‐G@LG with or without the NIR laser irradiation in 4T1 tumor‐bearing mice. g) IVIS images describing the intratumor retention time of DiR in free I/D‐G and I/D‐G@LG with or without the NIR laser irradiation in 4T1 tumor‐bearing mice. h) PD‐L1 expression of 4T1 tumor tissues after different treatments. i) Mean fluorescence intensity (MFI) of T_EX_ per tumor weight after different treatments. Data are shown as means ± SEM (n  =  4). j) Relative PD‐L1 expression of T_EX_ after different treatments. Data are shown as means ± SEM (n  =  4). The comparison of the two groups was followed by an unpaired Student's t‐test (two‐tailed). ^*^ *P* < 0.05, ^**^ *P* < 0.01 and ^***^ *P*<0.001.

We have designed a multistage drug carrier for the co‐delivery of hydrophobic exosome inhibitor SFX and immune checkpoint inhibitor aPDL1. In brief, a programmable LG was used as the primary‐stage carrier for all elements loading to realize an all‐in‐one strategy, PEG‐modified reduced graphene oxide nanosheets (NRGO) were chosen as the second‐stage carrier for SFX as well as the photosensitizer to control the drug release from LG to realize all‐in‐control strategy (**Figure** **3**a). First, NRGO was synthesized following procedures in Figure S1 (Supporting Information) and displayed a higher photothermal conversion efficiency than graphene oxide nanosheets (NGO) (Figure S2, Supporting Information). NRGO was subsequently used for SFX loading to obtain SFX‐loaded NRGO (S‐G). The particle size of the sulfisoxazole‐loaded NRGO (S‐G) was measured using dynamic light scattering (DLS), presenting an average diameter of 143.3 nm (Figure S3, Supporting Information). Additionally, the loading efficiency of SFX onto NRGO was quantified via HPLC analysis and was found to be ≈55.97%. The drug release from S‐G was accelerated when exposed to a laser or under an acidic microenvironment (Figure S4, Supporting Information). Then, a thermal‐responsive LG composed of PC and GDO was fabricated for the drug depot. The different mass ratios of PC/GDO were optimized to construct the drug depot and the PC/GDO ratio of 41/59 with gel‐to‐sol transition ability at ≈41 °C was chosen as the optimal formulation (Figure 3b; Figure S5, Supporting Information). S‐G and aPDL1 were subsequently loaded into the PC/GDO binary system through the “macrosol technique” 20. The lipid precursor could transform into gel quickly when injected into the aqueous phase and undergo reversible gel‐to‐sol transition as the temperature beyond 41 °C (Figure S6, Supporting Information). Meanwhile, the LG could remain in a gel state after three heating‐cooling cycles between 37 and 45 °C, which confirmed the reversible gel‐to‐sol transition ability of LG (Figure 3c).

### 2.3 Degradation Behavior and Biocompatibility Evaluation In Vitro and In Vivo

We next investigated the degradation behavior of LG in vitro and in vivo. For the in vitro study, LG was immersed in PBS with different concentrations of lipase, and the mass and appearance of LG were recorded at the predetermined time points. As shown in Figure S7 (Supporting Information) and Figure 3d, the gel started by absorbing water and swelling at the first several days, then the gel began erosion in a lipase‐concentration‐dependent manner. The degradation process was accelerated as the concentration of lipase increased. To evaluate gel degradation in vivo, the lipid precursor was injected subcutaneously to make a comparison of the degradation manner of the LG with or without laser irradiation (Figure S8, Supporting Information). It could be observed that the lipid precursor could quickly form a gel in situ within 1 h after injection and maintained under the skin. It was notable that the gel matrix could be gradually degraded with the volume of gel reduced and the laser irradiation could speed up the erosion process. To evaluate the biocompatibility of LG, skins around the injection sites were dissected at different time points, and hematoxylin‐eosin staining (H&E) images of these samples showed no inflammatory reaction (Figure S8, Supporting Information). The results of safety evaluation tests in vitro were in line with the in vivo biocompatibility evaluation (Figure S9, Supporting Information). As the MTT assay of gel leachates showed, all of the cell survival rates co‐cultured with different concentrations of extracted leachates exceeded 80%, suggesting no obvious toxicity existed. Indeed, both the in vivo and in vitro evaluation indicated that the designed drug carrier possessed good biocompatibility.

### 2.4 Photothermal Effect and Drug Release Behavior of LG In Vitro and In Vivo

Encouraged by the prominent NIR absorption ability of NRGO, the in vitro photothermal performance of NRGO‐loaded LG (NRGO@LG) was determined by an infrared thermal imaging camera (Figure S10, Supporting Information). A similar temperature upward trend could be observed from the NRGO solution group (Free NRGO) and NRGO@LG. Specifically, both the free NRGO group and NRGO@LG group displayed good photothermal conversion efficiency. It was worth noting that the temperature of the NRGO@LG group grew faster than the solution, which may be due to the accumulation and condensation of the gel matrix during the gel formation process. Furthermore, the in vivo photothermal conversion ability was also investigated in the tumor‐bearing BALB/c mice. When the volume of the tumors reached 50–100 mm^3^, each mouse was administered through intratumor injection with 50 µL of free NRGO solution and NRGO@LG, respectively. The blank LG group and PBS group were set as control groups. After laser irradiation, the local temperature of tumors increased significantly within 2 min and could be maintained at ≈45 °C (Figure 3e; Figure S11, Supporting Information). Taken together, the initial rapid rising temperature and the subsequent thermal stability ensured the accuracy of temperature control for the treatment requirement.

Owing to the reversible temperature‐responsive phase transition ability of LG, the LG will undergo a phase transition process upon heating, thereby affecting the drug release behavior. So, laser irradiation was also used as the trigger for drug‐controlled release. To validate our proposal, IgG was used as the alternative to aPDL1 and co‐loaded with S‐G into the LG to evaluate the photo‐triggered drug release manner in vitro (Figure S12, Supporting Information). The release of IgG and SFX was relatively stable without irradiation, suggesting LG was a promising drug depot for sustained drug release. Whereas, LG that accepted the laser irradiation at 4 and 48 h displayed a higher drug release rate, indicating the laser could effectively stimulate the enhanced drug release. The results showed that only 4.9% of IgG and 3.7% of SFX were released for LG without laser irradiation at 96 h, however, 8.6% of IgG and 4.5% of SFX were released for LG after irradiation twice. Meanwhile, IgG and SFX could be released from the LG sol form at a higher rate than from the LG gel form (Figure S13, Supporting Information). All of the above suggested that the gel‐to‐sol phase transition could accelerate the release of IgG and SFX, indicating the thermal effect could play a crucial role in the drug‐controlled release. Moreover, we also evaluated the drug‐release behaviors of the LG under different conditions. As displayed in Figure S14 (Supporting Information), the acidic condition could accelerate the drug release both of SFX and IgG. What's more, the drug release behaviors were tested after storage at −20 °C for seven days, no significant changes in drug release were observed in Figure S15 (Supporting Information), confirming the stability of our delivery system under prolonged storage conditions.

To investigate the in vivo release behavior of the drug‐loading LG with or without laser irradiation, Cy3 labeled IgG (IgG‐Cy3) was co‐loaded with Dir‐NRGO (D‐G) of which DiR was used as an alternative to SFX. The tumor‐bearing BALB/c mice were used for evaluation and the formulations were intratumorally injected. For the NIR group, the LG exposed to NIR for 4 times, and free IgG‐Cy3/ DiR‐NRGO solution (Free I/D‐G) was served as the control. To monitor the release of IgG‐Cy3 and DiR, IVIS was used to track the fluorescent signals at the predetermined time points. From the IVIS data (Figure 3f,g), the fluorescence of the free I/D‐G group decreased quickly in 2 days. Compared with the free group, both LG with or without irradiation showed a more durable drug release owing to the sustained effect of LG depot. In addition, laser irradiation also played a key role in accelerating the release of IgG‐Cy3 and DiR.

We also investigated the influence of mild PTT on TME. In our previous study, we confirmed that mild temperatures (37, 40, 43, and 45 °C) could cause an increase in the expression of PD‐L1 on the 4T1 cells in vitro.^[^ ^4a^ ^]^ We further studied the influence of mild heating on the expression of PD‐L1 in the tumor tissues. The mice breast cancer orthotopic model was used for in vivo investigation. When the tumor volume reached ≈50–100 mm^3^, 50 µL of NRGO@LG was injected intratumorally into different groups, and PBS was used as the control group. The photothermal groups were exposed to the laser at 0, 2, 4, and 6 day post administration and the different temperatures of different groups were controlled manually using an infrared thermal imaging camera. On the 8th day, the tumors were harvested for Western blot analysis and immunohistochemistry analysis. Compared with the untreated group, it could be easily observed that the expression of PD‐L1 was significantly promoted when the tumors were heated above 43 °C, which demonstrated that the heating could upregulate the expression of PD‐L1 (Figure 3h; Figure S16, Supporting Information). These results were in line with our results from in vitro experiments suggesting that the tumors upregulated the immunosuppressive factor for immune evasion.

Exosomes are vesicles containing bioactive molecules that have been proved to play important roles in cell‐to‐cell communication and immune response modulation.^[^ ^15^ ^]^ What's more, the former study demonstrated that the exosome secretion was increased in a temperature‐dependent manner,^[^ ^8^, ^16^ ^]^ which encouraged us to study the influence of mild PTT on the secretion of exosomes from tumor cells in vitro and in vivo. For the in vitro experiments, 4T1‐CD63‐GFP cells were cultured for further analysis. The cells were incubated for 24 h before the medium was collected for exosome isolation. The cells were harvested and lysed and the total proteins of cells were used for normalization. Exosome purification was carried out by the standard ultra‐centrifugation techniques and further characterization by nanoparticle tracking analysis (NTA), Western blot, and transmission electron microscopy (TEM). The size of exosomes was determined by NTA with the peak at 132 nm (Figure S17, Supporting Information). The TEM picture also confirmed the morphology of exosomes (Figure S18, Supporting Information). Western blot further confirmed the expression of common markers of exosomes including CD63 and Tsg101 suggesting the existence of exosomes (Figure S19, Supporting Information).

To understand the temperature effects on the secretion of exosomes, the cells were immersed in a water bath of different conditions (37 and 45 °C). After incubation for another day, the exosomes were collected for quantitative analysis. As the results showed in Figure S20 (Supporting Information) the secretion of exosomes was significantly increased with the temperature increased. To investigate the influence of the photothermal effect on the exosome secretion in vivo, 4T1‐CD63‐GFP cells were inoculated orthotopically for the tumor‐bearing model. NRGO@LG was intratumorally administered for photothermal effect and an increased temperature (45 °C) of tumor sites was maintained using laser irradiation. NRGO@LG without laser and PBS were used as control groups. The tumors were harvested for the isolation of exosomes. T_EX_ was first isolated according to the procedures established by others^[^ ^17^ ^]^ and the fluorescence specifically on T_EX_ was detected for quantitative analysis. The data showed that the mild PTT could significantly enhance the secretion of exosomes from tumor cells in vivo, which was in accordance with the in vitro results. Owing to the abundance of reports of the immunosuppression effects of exosomes, we hypothesized that mild PTT will facilitate the tumor cells with enhanced immunosuppression through increased exosomes secretion to realize the immune escape. So, SFX was chosen as the exosome inhibitor to reduce the exosome secretion as well as to eliminate the immunosuppression of exosomes. The exosome inhibition effects were checked in vitro and in vivo. When compared with the PBS group, SFX could significantly reduce the exosome secretion both in vitro and in vivo (Figure 3I; Figure S20, Supporting Information). Meanwhile, the inhibition effects were more effective as compared with the mild PTT group, suggesting that SFX treatment could effectively eliminate the enhanced exosome secretion induced by PTT. Meanwhile, we also monitored the expression of exosomal PD‐L1. As the results showed in Figure 3j, the exosomal PD‐L1 was increased, indicating the enhanced immunosuppressive signature of exosomes in TME after the mild PTT treatment. Moreover, we also observed that mild PTT similarly accelerated exosome release and increased the expression of PD‐L1 in the TME of human breast cancer models as it did in mouse 4T1 models. Importantly, the administration of SFX effectively reduced the secretion of these exosomes, aligning with our observations in the mouse model (Figures S21 and S22, Supporting Information). Taken together, mild PTT not only increases the number but also the immunosuppressive level of T_EX_. Thus, mild PTT along with exosome inhibition could realize better immune efficacy.

### 2.5 Antitumor Effect in a Mouse Breast Cancer Model

**Figure 4:**
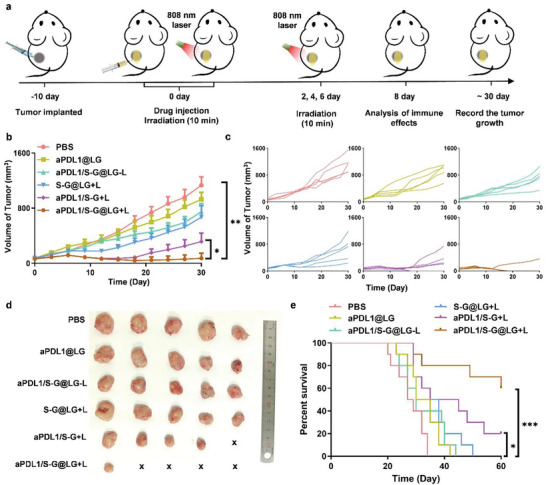
In situ gel depot for inhibition of 4T1 carcinoma tumor growth in vivo. a) Schematic illustration of the animal experimental design. b) Primary tumor growth curves with the mean tumor volumes of 4T1 tumor‐bearing BALB/c mice model. Data are shown as means ± SEM (n = 5). c) Primary tumor growth curves of individual mice in different groups of 4T1 tumor‐bearing BALB/c mice model. d) The tumor images were obtained from the tumor‐bearing BALB/c mice on day 30 after treatments. e) The survival percentages of the tumor‐bearing BALB/c mice (n = 10). The comparison of the two groups was followed by an unpaired Student's t‐test (two‐tailed). ^*^ *P* < 0.05, ^**^ *P* < 0.01 and ^***^ *P*< 0.001.

Based on the aforementioned experiment results, we wondered if our designed combination strategy was an effective anti‐tumor treatment in vivo. A mouse breast cancer orthotopic model was used for in vivo experiments. The tumor‐bearing BALB/c mice were randomly divided into 6 groups. When the volume of the tumors reached 50–100 mm^3^, each mouse was administered through intratumor injection with PBS, aPDL1@LG, SFX‐NRGO@LG (S‐G@LG), aPDL1/SFX‐NRGO solution (aPDL1/S‐G) and aPDL1/SFX‐NRGO@LG (aPDL1/S‐G@LG) containing SFX 20 µg and aPDL1 100 µg, respectively. NIR irradiation with 808 nm laser was applied to the mice in the groups of S‐G@LG, aPDL1/S‐G, and aPDL1/S‐G@LG 4 times on the days of 0, 2, 4, and 6 post administration in a pattern displayed in **Figure** **4**a. These groups that received the laser irradiation were depicted as S‐G@LG+L, aPDL1/S‐G+L, and aPDL1/S‐G@LG+L, whereas S‐G@LG‐L, aPDL1/S‐G‐L, and aPDL1/S‐G@LG‐L were used for these groups without irradiation. After administration, anti‐tumor effects were evaluated by monitoring the tumor size (Figure 4b–d). From the tumor growth curve of primary tumors, it could be observed that aPDL1/S‐G@LG+L group and aPDL1/S‐G+L group that co‐loaded with the exosome inhibitor SFX significantly slowed the growth of 4T1 tumors in comparison to the groups of aPDL1@LG, aPDL1/S‐G@LG‐L and S‐G@LG+L that just received a single treatment or without PTT treatment. Especially for the aPDL1/S‐G+L group, tumors of four mice were completely ablated and the tumor inhibition rate reached 94% on day 30. The aPDL1/S‐G+L group had a comparable tumor inhibition rate with the aPDL1/S‐G@LG+L group in the first two weeks of treatment, indicating that combination therapy could effectively activate the body's immune response. However, the therapeutic effect gradually disappeared two weeks later owing to the elimination of drugs, which demonstrated that a long‐lasting drug release scheme was necessary for exerting a long‐term treatment. When compared with these two groups above, only a low tumor inhibition rate of ≈35% was observed in the group of aPDL1/S‐G@LG‐L without external NIR irradiation. The LG depot remained a stable structure and the amount of drug released was not enough to alleviate the tumor immunosuppression owing to the lack of laser and heat, indicating that the laser irradiation was essential to regulate drug release, alleviate immunosuppression, and activate adaptive immunity. The S‐G@LG+L group displayed a moderate inhibition effect of tumor growth, but with the growth of the tumors and the enhancement of immunosuppression, a single treatment showed limited therapeutic effect on the tumors. Meanwhile, the aPDL1@LG group showed only weak tumor inhibition compared with the untreated group, suggesting that the therapeutic effects were limited.

Furthermore, the combination treatment strategy achieved superior survival benefits than other groups (Figure 4e). 60% of the mice in the group of aPDL1/S‐G@LG+L still survived on the 60th day after treatment. In addition, no noticeable weight loss or fluctuations were displayed for mice in each group, suggesting that there was no acute toxicity (Figure S23, Supporting Information). Further, no obvious histological or pathological changes were detected in the major organs including heart, liver, spleen, lung, and kidney, suggesting the biocompatibility of all formulations (Figure S24, Supporting Information). The above results suggested that this combined treatment strategy was essential to alleviate immune suppression and activate the body's adaptive immunity synergistically.

### 2.6 Activated Immunity in TME

To understand the underlying mechanism of the antitumor effects triggered by the combination strategy, immune cells and immune molecules in the tumors and lymph nodes (LNs) were analyzed.

**Figure 5:**
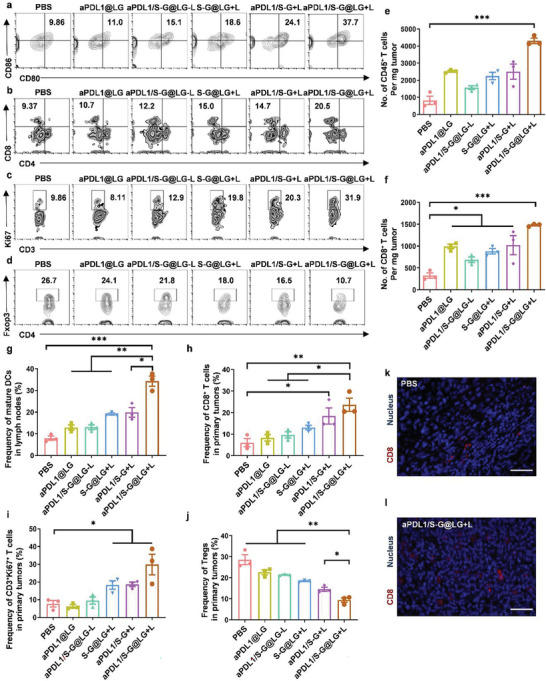
Activation of immune responses in TME and lymph nodes. a) Representative flow cytometric analysis of DCs maturation within the lymph nodes (gated on CD11c^+^ cells). b) Representative flow cytometric analysis of CD8^+^ T cell infiltration within the tumors (gated on CD3^+^ T cells). c) Representative flow cytometric analysis of CD3^+^Ki67^+^ T cell within the tumors (gated on CD45^+^ cells). d) Representative flow cytometric analysis of Tregs within the tumors (gated on CD4^+^ T cells). e) Absolute numbers of CD45^+^ lymphocytes in the primary tumors after different treatments were examined on day 8 after treatment. Data are shown as means ± SEM (n = 3). f) Absolute numbers of CD8^+^ T cells in the primary tumors after different treatments were examined on day 8 after treatment. Data are shown as means ± SEM (n = 3). g) DC maturation induced by different treatments on 4T1 tumor‐bearing mice in lymph nodes on day 8 after different treatments. (gated on CD11c^+^ cells). Data are shown as means ± SEM (n = 3). h) The frequency of CD8^+^ T cells in primary tumors after different treatments was examined on day 8 after treatment. Data are shown as means ± SEM (n = 3). i) The proliferation ability of T cells after different treatments within the tumors staining with CD3 and Ki67 examined on day 8 after treatment. Data are shown as means ± SEM (n = 3). j) The frequency of Tregs in primary tumors after different treatments examined on day 8 after treatment. Data are shown as means ± SEM (n = 3). k) Representative immunofluorescent staining image of tumor tissue after treatment with PBS. Blue: Nucleus; Red: CD8. Scale bar = 100 µm. l) Representative immunofluorescent staining image of tumor tissue after treatment with aPDL1/S‐G@LG+L. Blue: Nucleus; Red: CD8. Scale bar = 100 µm. The comparison of the two groups was followed by an unpaired Student's t‐test (two‐tailed). ^*^ *P*< 0.05, ^**^ *P*< 0.01 and ^***^ *P*< 0.001.

DCs are the most potent type of antigen‐presenting cell (APC), which play crucial roles in initialing and regulating adaptive immunities. Upon exposure to antigens, immature DCs will transform into mature DCs known as the upregulation of co‐stimulatory molecules CD80 and CD86. So, we first investigated the DC maturation in LNs. As the results showed in **Figure** **5**a,e, DC maturation of the aPDL1/S‐G@LG+L group was 4.3‐fold higher than that in the PBS group. Correspondingly, the DC maturation was also significantly improved in the treatment of aPDL1/S‐G, S‐G@LG+L, and aPDL1/S‐G@LG‐L groups, indicating the strong immune activation effect.

Recruitment of tumor infiltration lymphocytes (TILs) is associated with better clinical outcomes in many tumors.^[^ ^18^ ^]^ We further examined the immune microenvironment of the tumors after the combination therapy, the numbers of CD45^+^ lymphocytes, CD4^+^ T cells, and CD8^+^ T cells were significantly increased inside the tumors (Figure 5e,f; Figure S25, Supporting Information). The immunofluorescence of CD8^+^ T cells within the tumor of aPDL1/S‐G@LG+L group also illustrated the increased infiltration (Figure 5k–l). In addition, we also examined the frequency of the T lymphocyte subpopulation of the primary tumors. As shown in Figure 5b,f, the frequency of CD8^+^ T cells in aPDL1/S‐G@LG+L‐treated tumors was 2.3‐fold higher than that of the PBS group. Three groups receiving mild PTT could significantly increase the frequency of CD8^+^ T cells in the primary tumors, indicating the efficient recruitment of lymphocytes of mild PTT.

Besides the density or infiltration numbers of TILs in tumors, the activation state of TILs is correlated to the efficacy of immunotherapy.^[^ ^3^ ^]^ TILs promote anti‐tumor immunity via the production of proinflammation cytokines.^[^ ^19^ ^]^ The successful activation of TILs in response to anti‐tumor treatment was further confirmed by the increased production of interleukin 2 (IL‐2) and interferon γ (IFN‐γ) in tumors and serum of the aPDL1/S‐G@LG+L group, illustrating that the combination strategy could effectively induce the strong immune effects and cytotoxicity (Figures S26 and S27, Supporting Information). In addition, the proliferation ability of T cells was also used as an indicator of activation by monitoring Ki67.^[^ ^20^ ^]^ It was demonstrated in Figure 5c,i that the expression of Ki67 was significantly increased in the group of aPDL1/S‐G@LG+L, indicating the active proliferation status of T cells. Otherwise, regulatory T cells (Tregs) are a specialized subset of T cells known to inhibit antitumor effects. The accumulation of Tregs in tumors will lead to the immunosuppression of antitumor immune responses.^[^ ^21^ ^]^ We dramatically found that the combination treatments of the aPDL1/S‐G@LG+L group and aPDL1/S‐G+L group could effectively reduce the percentage of Tregs in the primary tumors by 78.1 and 62.0% respectively in the comparison with PBS, which were also much lower than those in the other treatments (Figure 5d,j). Moreover, Kyoto Encyclopedia of Genes and Genomes (KEGG) enrichment analysis (Figure S28, Supporting Information) further identified that the S‐G@LG+L group was enriched in multiple immune‐response associated functions, i.e., phagocytosis, positive regulation of cytokine production and T cell activation, indicating the synergistic role of exosome inhibition in activation of immune response.

Our data indicated that this combination treatment was able to enhance the stimulation of DCs, resulting in better priming of TILs and activation of T cells, which successfully sensitize the immunological “cold” tumor into “hot”, thus contributing to tumor eradication.

### 2.7 Combination Therapy for Treating Distal Tumors

**Figure 6:**
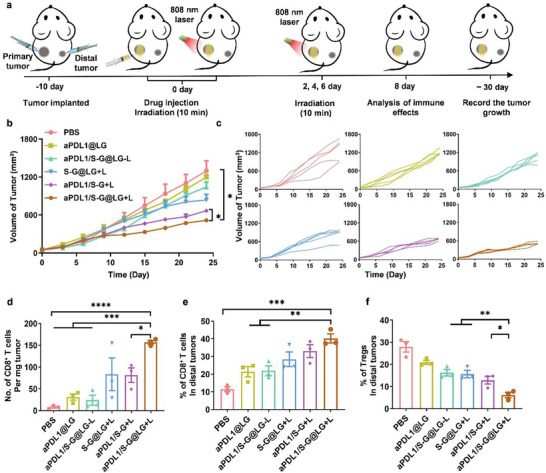
The therapeutic effect on distal tumors. a) Schematic illustration of the animal experimental design for distal tumors. b) Distal tumor growth curves with the mean tumor volumes of the 4T1 tumor‐bearing BALB/c mice model. Data are shown as means ± SEM (n = 5). c) Distal tumor growth curve of individual mice in different groups of 4T1 tumor‐bearing BALB/c mice model. d) Absolute numbers of CD8^+^ T cells in the distal tumors after different treatments were examined on day 8 after treatment. Data are shown as means ± SEM (n = 3). e) The frequency of CD8^+^ T cells in distal tumors after different treatments was examined on day 8 after treatment. Data are shown as means ± SEM (n = 3). f) The frequency of Tregs in distal tumors after different treatments was examined on day 8 after treatment. Data are shown as means ± SEM (n = 3). The comparison of the two groups was followed by an unpaired Student's t‐test (two‐tailed). ^*^ *P*< 0.05, ^**^ *P*< 0.01, ^***^ *P*< 0.001 and^****^ *P*< 0.0001.

With the confirmation that the combination strategy could effectively impede the growth of primary tumors and activate adaptive immunity, we investigated whether the local treatment with the combination strategy triggered the strong immune responses for the treatment of untreated distal tumors. The distal tumor model was established using mouse breast cancer cells. 4T1 cells were inoculated simultaneously with the primary tumors (**Figure** **6**a). Following the same treatment scheme for primary tumors as described above, the size of the distal tumor was monitored and recorded (Figure 6b,c). The growth rates of distal tumors in the groups of aPDL1/S‐G@LG+L and aPDL1/S‐G+L were significantly inhibited, whereas the single treatment groups such as S‐G@LG+L and aPDL1@LG showed limited efficacy on the inhibition of distant tumors, which suggested that only the combination therapy could efficiently activate the systemic immunity. A better tumor inhibition rate was achieved through a long‐term treatment using a sustained‐release LG depot.

To investigate the immune response in the distal tumors after various therapies, intratumor infiltration of lymphocytes was examined. The numbers of intratumor lymphocytes marked by CD45 and CD8 were all improved after combination treatment (Figure 6d; Figure S29, Supporting Information). In addition, by analyzing T cell subtypes, a significant increase of CD8^+^ T cells and a decrease of Tregs were observed in the group of aPDL1/S‐G@LG+L in Figure 6e,f, which has been reported to correct with a good prognosis in tumor treatment. The increased number of lymphocytes indicated a better inhibition effect for tumor growth.

### 2.8 Efficacy of Anti‐Metastasis and Long‐Term Immune Memory Effect

The occurrence of tumor metastasis is the major cause of cancer‐induced death, thus we are wondering if the combination treatment could activate a robust immune effect for anti‐metastasis. Encouraged by the excellent therapeutic effects of this combination strategy for primary and distal tumor inhibition as well as the improved survival of tumor‐bearing mice, we build a more aggressive whole‐body spreading tumor model to evaluate the anti‐metastasis effect by long‐term immune memory induced by the combination strategy.

**Figure 7:**
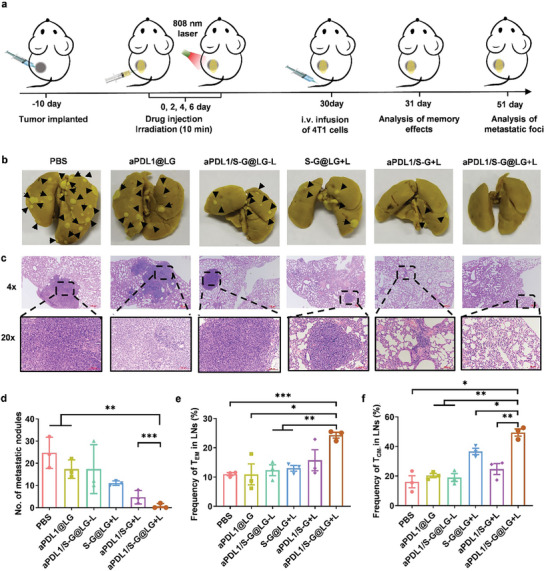
Metastasis prevention via long‐term immune effects. a) Schematic illustration for combination therapy‐mediated inhibition of tumor metastasis. b) Representative photographs of lung tissues with tumor metastasis. c) H&E staining of lung tissues was collected on day 51. d) Quantification of pulmonary metastasis nodules in different groups of 4T1 tumor‐bearing BALB/c mice. Data are shown as means ± SEM (n = 3). e) Proportions of T_EM_ cells in the lymph nodes were examined after 1‐day post intravenous infusion of 4T1 cells. Data are shown as means ± SEM (n = 3). f) Proportions of T_CM_ cells in lymph nodes were examined after 1‐day post intravenous infusion of the 4T1 cells. Data are shown as means ± SEM (n = 3). The comparison of the two groups was followed by an unpaired Student's t‐test (two‐tailed). ^*^ *P*< 0.05, ^**^ *P*< 0.01 and ^***^ *P*< 0.001.

The long‐term immune memory effect is an important feature of the adaptive immune response, which can help to quickly identify and recruit immune cells for elimination when exposed to the same antigen again. The model was established by injecting 4T1 cells through intravenous injection 30 days post‐treatment of the primary tumors. The lungs of each group were harvested for metastasis evaluation on the 21st day after the inoculation (**Figure** **7**a). The gross appearance of metastasis nodules revealed that each combination group receiving the mild PTT had a significant inhibition effect on lung metastasis when compared with the PBS group. As shown in Figure 7b, almost no lung metastatic nodules were observed on the lungs of mice in the aPDL1/S‐G@LG+L group, while the lungs of other groups were occupied with tumor nodules, especially the groups of PBS, aPDL1@LG, aPDL1/S‐G@LG‐L, and S‐G@LG+L. For further quantification, the aPDL1/S‐G@LG+L group substantially reduced lung nodules in comparison with the PBS group, 0.6 ± 1.2 lung nodules versus 24.6 ± 7.0 lung nodules (Figure 7d). The pulmonary metastasis nodules of mice were then stained using H&E for pathological analysis (Figure 7c). These results declared that this combined strategy could accommodate effectively ability of anti‐metastasis.

To explore the potential mechanism of tumor‐specific anti‐metastasis effect, the lymph nodes of mice were dissected 24 h after rechallenging by intravenous injection of 4T1 cells. Effector memory T cells (T_EM_) and central memory T cells (T_CM_) were analyzed at the cellular level by flow cytometry. When the antigens reappear, T_EM_ and T_CM_ can quickly induce immune responses and produce multiple cytokines for protection.^[^ ^22^ ^]^ In this study, we confirmed that the frequency of T_EM_ is significantly improved in the groups that received mild PTT treatment, which was in accordance with the results of statistical numbers of metastasis nodules. What's more, the aPDL1/S‐G@LG+L group showed the highest frequency of T_EM_ and T_CM_ in lymph nodes (Figure 7e,f; Figure S30, Supporting Information), indicating that the combined strategy could generate more‐potent antitumor immune effects.

## 3 Discussion

The TME plays an important role in the outcome of cancer treatments.^[^ ^23^ ^]^ Researchers have discovered that the intricate and diverse TME exerts a significant influence on the efficacy of ICB therapy. This complex microenvironment involves the expression of suppressive molecules, infiltration and activation status of T cells, secretion of suppressive cytokines, and the presence of extracellular vesicles carrying suppressive molecules.^[^ ^24^ ^]^

Mild PTT plays a pivotal role in altering the TME by enhancing tumor perfusion and facilitating the trafficking of immune cells. Importantly, mild PTT can also initiate immunogenic cell death, leading to an increased release of tumor antigens. These antigens are then captured by antigen‐presenting cells (APCs), such as dendritic cells, which play a key role in initiating a robust T‐cell‐mediated immune response. This process not only activates and proliferates T cells but also helps to elicit a specific anti‐tumor immune response. Thus, mild PTT serves as a critical modulator of immune activation, enhancing the efficacy of subsequent therapies by priming the immune system.^[^ ^25^ ^]^

In our previous study, we investigated the combination of mild PTT and ICB to enhance the recruitment of TILs and convert immunologically “cold” tumors into “hot” tumors. However, there are remaining issues that need to be addressed. First, it is unclear whether TILs recruited by mild PTT are in an activated or exhausted state. Second, our investigations have revealed that mild PTT enhances tumor immunosuppression by upregulating the expression of PD‐L1 on tumor cells and increasing exosome secretion. In particular, exosomes carrying surface PD‐L1 molecules not only facilitate intercellular communication but also trigger an immune checkpoint response, leading to systemic immune suppression upon release into the circulation, thereby promoting immune evasion by tumor cells. Given the critical roles of exosomes, inhibiting exosome secretion represents a promising strategy for overcoming the tumor‐suppressive environment and enhancing the antitumor immune response.

Therefore, this strategy holds promise in enhancing lymphocyte infiltration, alleviating immune system suppression, and converting “cold” tumors to “hot” tumors, thereby improving systemic immune response and achieving enhanced anti‐tumor efficacy. In light of these considerations, a rational design combining mild PTT and exosome inhibition with ICB treatment is proposed. This combination can enhance lymphocyte infiltration, and inhibit the upregulation of immunosuppressive PD‐L1 expression by exosome secretion, thereby improving the efficacy of ICB treatment.

In this study, a multistage drug carrier system was employed for combination therapy. The PC/GDO LG scaffold served as the primary drug depot for photosensitizers, exosome inhibitors, and antibodies, while NRGO acted as both a secondary drug carrier and a photosensitizer. The multistage LG scaffold was developed as a platform for the controlled and long‐term release of SFX and aPDL1. Both in vitro and in vivo drug release profiles demonstrated that drug release could be manually controlled through on‐demand external laser irradiation. Furthermore, the laser not only triggers drug release but also induces a mild photothermal effect, promoting lymphocyte infiltration.

Exosomes have emerged as important mediators of cellular communication and have been recognized as emerging players in the systemic regulation of tumor progression. SFX was identified as a novel inhibitor of exosome secretion from breast cancer cells. Our current investigations further demonstrated that SFX effectively reduces exosome secretion in vitro and in vivo, particularly when combined with mild photothermal therapy. The combination strategy has proven successful in modulating the tumor's immunosuppressive microenvironment to some extent, as revealed by immune mechanisms. Furthermore, this combination immunotherapy not only significantly increases lymphocyte infiltration but also confers T cells in TME with potent tumor‐killing abilities and enhanced proliferation capacity, ultimately resulting in a robust anti‐tumor immune response.

Above all, this combination immunotherapy possesses the characteristic of being easy to dose. It can be manipulated to achieve effective tumor inhibition, improve survival rates, prevent distant tumor growth, and establish a durable immune memory effect to guard against tumor relapse. Moreover, in advancing our combination therapy strategy, we acknowledge the need for further refinement in drug release mechanisms to ensure sustained efficacy and treatment specificity throughout the therapeutic regimen. Future studies will focus on optimizing the release kinetics to maintain consistent therapeutic levels over extended periods. Moreover, while our current results are promising, the long‐term efficacy and safety of this strategy will be rigorously evaluated in diverse animal models.

## 4 Experimental Section

### Materials

Soybean phosphatidylcholine (PC) and glycerol dioleate (GDO) were obtained from Nanjing Well Pharmaceutical Co., LTD. (Nanjing, China). Graphene oxide (GO) was purchased from XFNANO Materials Tech Co., LTD. (Nanjing, China). All tool compounds were used as obtained. Sulfisoxazole (SFX) with a purity of 99.95% was purchased from MCE (Shanghai, China). aPDL1 (catalog number BE0101) used in vivo was purchased from BioXCell (West Lebanon, NH, USA). Fixable viability stain 780 (catalog number 565388), anti‐CD45‐FITC (catalog number 553080), anti‐CD3‐BB700 (catalog number 566494), anti‐CD4‐BV605 (catalog number 563151), anti‐CD8‐PE‐CY7 (catalog number 552877), anti‐CD25‐APC (catalog number 557192), anti‐CD279‐BV421 (catalog number 562584), anti‐Ki67‐BV650 (catalog number 563757), anti‐CD11c‐PE‐CY7 (catalog number 558079), anti‐I/A‐I‐E‐Percp‐Cy5.5 (catalog number 562363), anti‐CD80‐PE (catalog number 553769), anti‐CD86‐APC (catalog number 558703), anti‐CD4‐Percp‐Cy5.5 (catalog number 566407), anti‐CD8‐PE‐CY7 (catalog number 552877), anti‐CD44‐PE (catalog number 553134), anti‐CD62L‐APC (catalog number 553152) and transcription factor buffer set (catalog number 562574) were obtained from BD Biosciences (San Jose, USA). Anti‐FOXP3‐PE (catalog number 12‐5773‐82) was purchased from Invitrogen (Carlsbad, USA). The primary antibodies for Western blot analysis: anti‐CD63 (catalog number 67605‐1‐Ig) and anti‐TSG101 (catalog number 28283‐1‐AP) were purchased from Proteintech Group (Chicago, USA).

### Cell Lines

The human umbilical vein endothelial cell line (HUVEC), metastatic murine breast cancer cell line (4T1), human breast cancer cell line (MDA‐MB‐231), and mouse macrophage cell line (RAW264.7) were purchased from the American Type Culture Collection (ATCC). The HUVEC, MDA‐MB‐231, and RAW264.7 cell lines were maintained in Dulbecco's modified Eagle's medium (DMEM; Hyclone, Cytiva) with 10% fetal bovine serum (FBS, Royacel, China), 100 U mL^−1^ penicillin/streptomycin and 1% L‐glutamine in an incubator at 37 °C in 5% CO_2_. 4T1 cells were cultured in Roswell Park Memorial Institute (RPMI) 1640 medium with the same supplementary as the other cells.

### Animals

BALB/c mice (6–8 weeks, 18–22 g) were purchased from the Junke biological Co., LTD. (Nanjing, China). All animal studies were carried out following the National Institute of Health Guide for the Care and Use of Laboratory Animals and approved by the Animal Ethics Committee of China Pharmaceutical University (Ethics number: 20201019).

### Preparation and Characterization of NRGO and SFX‐Loaded NRGO (S‐G)

NRGO was prepared using GO and PEG‐modified poly (maleic anhydride‐alt‐1‐octadecene) (PMHC_18_‐mPEG_5000_). First, PMHC_18_‐mPEG_5000_ was synthesized using methoxy poly(ethylene glycol)‐amine (PEG_5000_‐NH_2_, Ponsure biological), poly(maleic anhydride‐alt‐1‐octadecene) (PMHC_18_, molecular weight 30000–50000 Da) and EDC·HCl. Briefly, PMHC_18_ 285.7 mg and PEG_5000_‐NH_2_ 10 mg were dissolved in DMSO/Pyridine (9:1), then EDC·HCl 21.8 mg was added into the mixture and stirred for another 24 h. After the reaction, the mixture was dialyzed with 10 kDa MWCO to remove the excess PMHC_18_, then the product was obtained by lyophilized.

For reduced graphene oxide (RGO), GO (50 mg) was added into 10 mL of ultra‐pure water for sonication to prepare GO suspension. The GO suspension was placed into the oil bath at 90 °C for 24 h to obtain RGO. The obtained RGO suspension was lyophilized to obtain RGO powder.

To prepare NRGO, RGO (50 mg) was added into 10 mL ultra‐pure water and sonicated for 30 min to prepare RGO suspension. Then the excess of PMHC_18_‐mPEG_5000_ was added, and the mixture was sonicated for another 2 h to prepare NRGO. The prepared nanoparticles were ultra‐centrifugated and rinsed 5 times to remove excess polymers, then re‐suspended into water and filtered through a 0.22 µm filter to obtain NRGO nanoparticles.

For the preparation of sulfisoxazole‐loaded NRGO (S‐G), SFX dissolved in 100 µL DMSO was added into the NRGO solution at a mass ratio of 2:1, sonicated in the ice bath for 30 min then stirred for 12 h at room temperature. The excess drug was removed by centrifugation. The amount of SFX loaded onto the NRGO was determined via high‐performance liquid chromatography (HPLC) using an Ultimate 3000 HPLC system (ThermoFisher, Waltham, USA). Chromeleon 7 software was used for process monitoring, data acquisition, and system control. A reverse phase C18 column (ZORBAX SB C18, 5 µm, 250 mm × 460 mm, Agilent, Santa Clara, USA) was used for separation at the temperature of 30 °C. The mobile phase consisted of distilled water (A) and acetonitrile (B) with 0.1% formic acid, respectively. The mobile phase flowed at the rate of 1 mL min^−1^ with the gradient elution. The total running time was set as 20 min. The initial condition was set at 85% A with a linear gradient to 55% over 14 min, followed by from 55 to 5% until 16 min, and went back to 85% at 16 min and 30 s. SFX was detected at 280 nm by a UV detector.

The particle size and zeta potential of nanoparticles were characterized using a BeNano 180 Zeta nanosizer (Bettersize Instruments, China) and a Malvern Zeta Sizer Nano series (Malvern Instruments, Worcestershire, UK).

### Preparation of the aPDL1/S‐G@LG

The drug‐loaded LG samples were prepared via the “Macrosol” technique according to the previous study.^[^ ^26^ ^]^ In brief, PC (100 mg), aPDL1 (2 mg), and S‐G (containing SFX 20 µg) were added into 0.5 mL of distilled water containing 3% sucrose. The mixture was mixed sufficiently and lyophilized. The oil phase precursor of LG was prepared by mixing appropriate amounts of PC and GDO with ethanol (10%). Then, 1 mL of the lipid precursor was introduced into the lyophilized powder system mentioned above. The non‐aqueous system was then mixed in a rolling mixer at room temperature to make a homogenous lipid formulation. The LG preparation was done under sterile conditions. All sample compositions herein were calculated by weight.

### Rheological Test

Rheology experiments were carried out using a DHR‐2 dynamic shear rheometer (TA Instruments, USA) equipped with a 20 mm parallel plate geometry. The prepared LG (200 mg) was placed on the middle of the plate and allowed to be equilibrated for 5 min before the rheological test. The frequency sweep tests were carried out within the range of 0.1–100 Hz with the strain as 1% at room temperature. The storage modulus (G’) and loss modulus (G’’) were recorded as a function of frequency.

For the determination of phase transition temperature (T_g_), temperature sweep experiments were carried out within the temperature range of 25—80 °C with the angular frequency (ω) as 6.28 rad s^−1^ and strain (γ) as 1%. G’ and G’’ were recorded and the temperature of the intersection point was regarded as T_g_. All the experiments were conducted within the linear viscoelastic region.

### In Vitro LG Degradation Study

The LG precursor (50 µL) was injected into the PBS at a pH of 7.4 containing different concentrations of lipase (0, 2, 5, and 10%). All the samples were placed at 37 °C during the whole process. The LG was taken out at predetermined time points and recorded the weight of the remaining samples. The morphology changes were also recorded by photographing.

### In Vitro Drug Release Study

IgG/S‐G@LG (50 µL) containing IgG (3 mg), SFX (100 µg), and NRGO (180 µg) was used for drug release study. The samples were divided into three groups, LG with/without laser irradiation that incubated at 37 °C and LG that incubated at the temperature above the phase transition temperature. 2 mL of PBS (pH 7.4) was used as release medium. At a specified timepoint interval, a 1 mL aliquot was taken out and replaced with fresh PBS. Each sample of the laser irradiation group was irradiated with an 808 nm laser to keep the temperature at 45 °C for 10 min. The released amount of IgG was determined using a BCA kit (Beyotime Biotechnology) and the released amount of SFX was determined using HPLC.

### Cytotoxicity Evaluation In Vitro

The LG was immersed and extracted in the DMEM medium for 24 h to obtain the leachates. A series of dilution solutions were prepared to obtain various concentrations of the leachates (100, 50, 25, 12.5, and 6.25%). Cytotoxicity evaluation was conducted using the MTT [3‐(4,5‐dimethylthiazol‐2‐yl)‐2,5‐diphenyltetrazolium bromide] assay. 4T1, HUVEC, and RAW264.7 cell lines were seeded in 96‐well plates and exposed to different concentrations of leachates for 24 and 48 h. At the end of the incubation, 0.5 mg mL^−1^ of MTT was added into each well and incubated for another 4 h. Then, 200 µL of DMSO was added to dissolve formazan crystals. The absorbance was measured at 490 nm by a microplate reader (POLARstar Omega, BMG LABTECH, German).

### Isolation and Characterization of Exosomes

For the in vitro experiment, the tumor cell supernatant was collected and processed using differential ultracentrifugation reported by Théry et al.^[^ ^27^ ^]^ Briefly, the supernatant was centrifuged at 300 × g for 10 min, 2000 × g for 10 min, and 10 000 × g for 30 min in sequence. Then the final supernatant was ultracentrifuged at 100 000 × g for 70 min to pellet the exosomes. The pellets were resuspended with PBS and ultracentrifuged at the same speed again. The purified exosomes were resuspended with PBS for further analysis. The purified exosomes were characterized using Western blot, Nanoparticle tracking analysis (NTA), and transmission electron microscopy (TEM).

For the Western blot analysis, 4T1 cells were lysed with RIPA buffer (P0013B, Beyotime, Jiangsu, China) supplemented with 1 mm PMSF (ST506, Beyotime, Jiangsu, China). The analysis of exosomes was performed as described previously. In brief, the proteins were first separated by SDS‐PAGE and subsequently transferred onto PVDF membranes. After the blockade of 5% defat milk, the primary antibodies were added and incubated at 4 °C overnight. The PVDF membranes were washed with PBST (PBS with 0.1% Tween 20). The secondary antibodies were incubated for 1 h at room temperature under a slight shake. Then, the bands of proteins were observed using the ECL kit (Enhanced Chemiluminescence kit HRP, 20210609, Fdbio, China).

For the NTA analysis, the purified exosomes were analyzed using a Zetaview (Particle Metrix, Meerbusch, Germany) and its corresponding software (ZetaView 8.05.04). For analysis, 1 mL of each sample diluted in ultra‐pure water was loaded into the detection cell. The instrument measured each sample at 11 different positions and the size distribution was calculated by the software.

TEM was used to observe the morphology of exosomes. The purified exosomes were negatively stained with phosphotungstic acid.

### In Vitro Evaluation of Exosome Release and Inhibition

The 4T1‐CD63‐GFP cell line was used for exosome release evaluation. For photothermal effect induced exosome release assay, the cell was incubated under a 45 °C water bath for 10 min. Then the medium was refreshed with DMEM medium containing 10% exosome‐depleted FBS for another 24 h incubation. For the SFX inhibition assay, cells were treated with different concentrations of SFX (100 µm) for 24 h under the medium containing 10% exosome‐depleted FBS. At the end of the time point, the cell culture supernatant was collected and fluorescence intensity was determined using a fluorescence microplate reader (EnVision, PerkinElmer, USA) at 488 nm (excitation) and 530 nm (emission) for exosome release analysis.

### In Vitro Evaluation of Photothermal Effect

To investigate the photothermal effect of NRGO@LG, blank LG, free NRGO, NRGO@LG and were added into the Eppendorf tubes. The samples were irradiated with an 808 nm laser for 10 min at the initial power density of 1 W cm^−2^, the laser power density was manually controlled to maintain the temperature at 45 °C. The blank LG and PBS were conducted in the same conditions as the control. The temperature was recorded every 30 s by an infrared thermal camera (FLIR E50, USA) to obtain the photothermal heating curves.

### In Vivo Evaluation of Photothermal Effect

To evaluate the photothermal effect in vivo, the tumor model was established first. The mice breast cancer models were established on female BALB/c mice by inoculating 1 × 10^6^ of 4T1 cells orthotopically into the 4th mammary pad of mice to grow a tumor. 50 µL of NRGO@LG and Free NRGO was injected into the tumor when the tumor volume reached ≈50 mm^3^. The tumor sites were irradiated with an 808 nm laser for 10 min. The blank LG and PBS were conducted in the same conditions as the control. The temperature was recorded every 30 s by an infrared thermal camera (FLIR E50, USA) to obtain the in vivo photothermal heating curves.

### In Vivo Evaluation of Exosome Release and Inhibition

To evaluate the exosome release of tumor cells in vivo, the mouse breast cancer model was established as described above. 4T1‐CD63‐GFP cells were used to grow a tumor. After the tumor volume reached 50 mm^3^, 50 µL of NRGO@LG and S‐G@LG were injected into the tumor. For the irradiation groups, the tumor sites were irradiated using an 808 nm laser, and the temperature of tumor sites was maintained at 45 °C for 10 min at determined timepoints, i.e., 4 h, 2, 4, and 6 days after the administration. The tumor tissues were harvested on 8th day. 50 µL of PBS served as the control group. The tumors were weighted accurately before further processing.

The isolation of T_EX_ was carried out according to the protocols reported previously.^[^ ^17^ ^]^ In brief, tumors were cut into small pieces, appropriate volume of collagenase D (2 mg mL^−1^) and DNase I (40 U mL^−1^) was added and incubated at 37 °C for 30 min. After digestion, the mixtures were filtered through a 70 µm cell strainer. Then the filtrate was collected and followed by the processes of differential centrifugation described above for exosome isolation. Fluorescence intensity determined by the fluorescence microplate reader was normalized by tumor weight, which served as the index for the evaluation of exosome release and inhibition. Furtherly, for the determination of PD‐L1 on exosomes, the total exosomal proteins were first determined by BCA assay, and 200 µg of the total exosomal proteins were used for exosomal PD‐L1 determination using a PD‐L1 ELISA kit (Cusabio, China).

To evaluate the exosome release of tumor cells in a human breast tumor model, human breast cancer cells (MDA‐MB‐231) xenografted into Balb/c nude mice. After the tumors reached a volume of 50 mm^3^, 50 µL of NRGO@LG and S‐G@LG was administered directly into the tumors. These treatments were complemented by controlled irradiation using an 808 nm laser to maintain the tumor site temperature at 45 °C for 10 min at specific intervals (4 h, 2, 4, and 6 day post‐administration), with PBS serving as a control. The tumors were weighted accurately before further processing. Exosomes in the TME were isolated for further analysis.

### In Vivo Evaluation of LG Degradation

To evaluate the LG degradation in vivo, 50 µL of NRGO@LG was injected subcutaneously with or without laser irradiation. For the irradiation group, the 808 nm laser was irradiated four times at the 4 h, 2, 4, and 6 days post‐injection at 45 °C for 10 min. The mice were sacrificed and photographs of the remaining LG in vivo were taken. Furthermore, the skin around the LG was dissected and examined by the H&E staining for biocompatibility evaluation.

### In Vivo Drug Retention Study

To evaluate the intratumor drug retention of SFX and aPDL1, DiR was selected as the model fluorescence molecule for SFX, and Cy3‐labeled IgG was selected to mimic the antibody. Cy3‐IgG/DiR‐NRGO@LG (I/D‐G@LG) was injected into the tumor with or without laser irradiation. And free Cy3‐IgG/DiR‐NRGO‐PEG (Free I/D‐G) was injected into the tumor as control. Fluorescence was monitored using the IVIVS Spectrum In Vivo Imaging System (Perkin Elmer, USA) and analyzed with Living Imaging software (Perkin Elmer, USA).

### Antitumor Study on 4T1 Mouse Model

To study the antitumor effects of combination therapy, a mice breast cancer model was used for the evaluation. 1 × 10^6^ of 4T1 cells were inoculated orthotopically into the fourth mammary pad of mice. The mice were randomly divided into 6 groups when the primary tumor volume reached ≈50–100 mm^3^. For administration, the mice were administered with 50 µL of free aPDL1/SFX‐NRGO (aPDL1/S‐G), aPDL1@LG, SFX‐NRGO@LG (S‐G@LG), aPDL1/SFX‐NRGO@LG (aPDL1/S‐G@LG) (containing SFX 20 µg, aPDL1 100 µg and NRGO 36 µg) through intratumor injection. The same volume of PBS was also injected into the tumor to serve as a control group. For photothermal groups, the 808 nm laser was irradiated at the appropriate power density to ensure the temperature at ≈45 °C for 10 min at 4 h, 2, 4, and 6 days. In the following days, the tumor volume and body weight of each mouse were recorded from the first day of administration until the end of the experiment. On the 30th day, 5 mice of each group were sacrificed, tumors were dissected for imaging, and major organs including heart, liver, spleen, lung, and kidney were harvested for H&E staining. For the survival curve of each group, the survival time of each mouse was recorded until the 60th day of the administration.

### Inhibition of Distant Tumor Growth

For the distant tumor model, the distal tumors were inoculated simultaneously with the primary tumors by injecting 5 × 10^5^ of 4T1 cells on the opposite side of the primary tumors. The mice were randomly divided into 6 groups when the primary tumor volume reached ≈50–100 mm^3^. Then the primary tumors were treated the same. The distal tumors were monitored and the volume of tumors was recorded from the first day of administration until the 30th day of treatments.

### Anti‐Metastasis Study

For the metastasis model, 4T1 cells were used for tumor model establishment and the primary tumors were inoculated as described above. After the primary tumor volume reached 50–100 mm^3^, the mice were randomly divided into 6 groups and followed the same treatment. 30 days post‐administration, each mouse was intravenously infused with 1 × 10^5^ of 4T1 cells. Three mice were sacrificed and their lymph nodes and spleens were harvested for memory T cells on the 30th day post‐administration. The other mice were fed until day 51, the lungs were dissected and fixed in Bouin's solution. The metastasis nodules were easy to observe and counted. The metastasis nodules were also checked by H&E staining.

### Sample Preparation for scRNA‐Seq

Fresh tumors were harvested and stored in GEXCOPETM tissue preservation solution (Singleron, China) before dissection. Tumors of three mice were dissociated and pooled together to generate the single‐cell suspension. First, the specimens were washed with PBS and cut into 1–2 mm pieces. Then, the tissue pieces were digested with GEXSCOPETM tissue dissociation solution (Singleron, China) at 37 °C for 30 min with agitation. Next, the mixtures were filtered through 70 µm cell strainers for single‐cell suspension and washed with PBS. GEXSCOPETM red blood cell lysis buffer (Singleron, China) was added at room temperature and incubation for 15 min to remove red blood cells. The solution was then centrifuged at 500 × g for 5 min and re‐suspended in PBS followed by cell sorting with FACS Fusion sorter (BD bioscience, USA). Alive CD45^+^ immune cells were sorted and purity evaluated. The overall cell viability was confirmed by trypan blue exclusion, which needed to be above 85%.

### scRNA‐Seq Library Preparation and Sequencing

Single‐cell suspensions (1 × 10^5^ cells mL^−1^) with PBS were loaded into microfluidic devices using the Singleron Matrix Single‐Cell Processing System (Singleron, China). Then, the scRNA‐seq libraries were constructed according to the protocol of the GEXSCOPE Single Cell RNA Library Kits (Singleron, China). Individual libraries were diluted to 4 nm and pooled for sequencing, which was sequenced on Illumina HiSeq X with 150 bp paired‐end reads.

### Sample Preparation and Sequencing for Bulk RNA‐Seq

Fresh tumors were dissected and lysed in TRIzol reagent followed by total RNA extraction. Purified RNA was processed with poly T‐mRNA capture before cDNA synthesis. Sequencing libraries were generated using the NEBNext Ultra RNA Library Prep Kit for Illumina (NEB, USA, Catalog: E7530L) following the manufacturer's recommendations. The qualified libraries were pooled and sequenced on Illumina platforms with the PE150 strategy in Novogene Bioinformatics Technology Co., Ltd (Beijing, China).

### RNA‐Seq Quality Control and Gene Quantification

The raw RNA‐Seq data were processed using Trimmomatic (v0.38) to remove low‐quality reads and potential adaptor contaminations with the default parameter. FastQC (v0.11.8) was used to visualize the quality of processed RNA‐Seq data. Then the trimmed reads were aligned to the mouse reference genome assembly build GRCm38 (mm10) using hisat2 (v2.0.5) with default parameters. The mapped reads were annotated to gencode gene exons (gencode.vM28.annotation_tran.gtf) and counted for each gene by feature count (v1.6.0).

### Differential Gene Expression Analysis and Enrichment Analysis

Differentially expressed genes in a given cell type compared with all other cell types were determined with the FindAllMarkers function from the Seurat package (Wilcoxon rank sum test, p values adjusted for multiple testing using the Bonferroni correction). Genes that expressed in more than 10% of the cells in both of the compared groups and with an average log (Fold Change) value greater than 0.25 were selected as differentially expressed genes (DEGs). Differentially expressed genes were filtered by fold change > 0.25 and *p*‐value < 0.05. For the differential expressed genes of different cell types, genes with *p*‐value < 0.05 and logFC > 0.25 were selected for enrichment analysis. The up‐regulation genes and down‐regulation genes were analyzed separately. The GO and KEGG enrichment analyses were performed by clusterProfiler (3.16.1).

### Cytokine Analysis

Samples of serum and tumor tissues were collected for cytokine analysis. For the extraction of cytokines from tumor tissues, the tumors were weighted accurately, cut into pieces, and transferred into the tissue homogenizer with 1 mL of PBS. The extract was centrifugated at 10 000 × g for 10 min and the supernatant was collected for further analysis. IFN‐γ, IL‐2, IL‐6, and TNF‐α were determined with ELISA kits according to the manufacturer's instructions (4A Biotech, China).

### Flow Cytometry

Flow cytometry was performed on a CytoFlex S flow cytometer (Beckman, USA). All the antibodies, buffers, and Truecount Tubes (BD, catalog number 340334) were used according to the manufacturer's instructions. The tumors and lymph nodes were harvested from each group of mice and single‐cell suspensions were collected for further analysis. The collected cells were incubated with fluorescence‐labelled antibodies for staining. The lymphocytes collected from tumors were incubated with Fixable viability stain 780 (BD, catalog number 565388), anti‐CD45‐FITC (BD, catalog number 553080), anti‐CD3‐BB700 (BD, catalog number 566494), anti‐CD4‐BV605 (BD, catalog number 563151), anti‐CD8‐PE‐CY7 (BD, catalog number 552877), anti‐CD25‐APC (BD, catalog number 557192), anti‐CD279‐BV421 (BD, catalog number 562584), anti‐ki67‐BV650 (BD, catalog number 563757) and anti‐FOXP3‐PE (Invitrogen, catalog number 12‐5773‐82,). DCs from lymph nodes and spleens were stained with Fixable viability stain 780 (BD, catalog number 565388), anti‐CD45‐FITC (BD, catalog number 553080), anti‐CD11c‐PE‐CY7 (BD, catalog number 558079), anti‐I/A‐I‐E‐Percp‐Cy5.5 (BD, catalog number 562363), anti‐CD80‐PE (BD, catalog number 553769) and anti‐CD86‐APC (BD, catalog number 558703). Memory T cells were stained with Fixable viability stain 780 (BD, catalog number 565388), anti‐CD8‐PE‐CY7 (BD, catalog number 552877), anti‐CD44‐PE (BD, catalog number 553134) and anti‐CD62L‐APC (BD, catalog number 553152).

### Statistical Analysis

All the data were presented as mean ± s.e.m. Student's *t*‐test was used for two‐group comparisons. One‐way ANOVA was used for multiple‐group analysis. GraphPad Prism 8 (GraphPad, USA) was used for statistical analysis. ^*^P < 0.05 was considered significant. ^**^P < 0.01 and ^***^P < 0.001 were highly significant compared to the corresponding control.

## Conflict of Interest

The authors declare no conflict of interest.

## Supporting information



Supporting Information

## Data Availability

The data that support the findings of this study are available from the corresponding author upon reasonable request.
