# Supplementary material for: Local Exosome Inhibition Potentiates Mild Photothermal Immunotherapy Against Breast Cancer
Source: Adv Sci (Weinh). 2024 Nov 22;12(2):2406328. doi: 10.1002/advs.202406328 (PMC11727390; doi:10.1002/advs.202406328)
Supplement: Supplementary file 1 — Supporting Information [file ADVS-12-2406328-s001.docx]

**Supplementary information**

Local exosome inhibition potentiates mild photothermal immunotherapy against breast cancer

Qian Chen^1,2,3,4,#^, Yanan Li^5,#^, Jiameng Hu^6,7,#^, Zhenyu Xu^1,2,3,#^, Shengyi Wang^1,2,3^, Naicong Cai^1,2,3^, Mengjiao He^1,2,3^, Yifang Xiao^1,2,3^, Yuan Ding^8^, Mengjuan Sun^1,2,3^, Chunjiayu Li^1,2,3^, Yiyang Cao^1,2,3^, Zhongyuan Wang^1,6^, Fang Zhou^1,4^, Guangji Wang^1,4^, Chen Wang^1,6,*^, Jiasheng Tu^1,2,3,*^, Haiyang Hu^1,6,9,*^, Chunmeng Sun^1,2,3,*^

1 State Key Laboratory of Natural Medicines, China Pharmaceutical University, 639 Longmian Avenue, Nanjing 211198, China.

2 Department of Pharmaceutics, School of Pharmacy, China Pharmaceutical University, 639 Longmian Avenue, Nanjing 211198, China.

3 NMPA Key Laboratory for Research and Evaluation of Pharmaceutical Preparations and Excipients, China Pharmaceutical University, 24 Tong Jia Xiang, Nanjing 210009, China.

4 Jiangsu Provincial Key Laboratory of Drug Metabolism and Pharmacokinetics, China Pharmaceutical University, 24 Tong Jia Xiang, Nanjing 210009, China.

5 School of Food and Pharmaceutical Engineering, Nanjing Normal University, No. 1 Wenyuan Road, Nanjing 210023, China.

6 School of Life Science and Technology, China Pharmaceutical University, 639 Longmian Avenue, Nanjing 211198, China.

7 Institute of Systems and Physical Biology, Shenzhen Bay Laboratory, Shenzhen 518055, China.

8 Department of Pharmaceutics, School of Pharmacy, Fudan University, Shanghai 201203, China

9 Central Laboratory, Shanghai Pulmonary Hospital, Tongji University School of Medicine, Shanghai 200433, China.


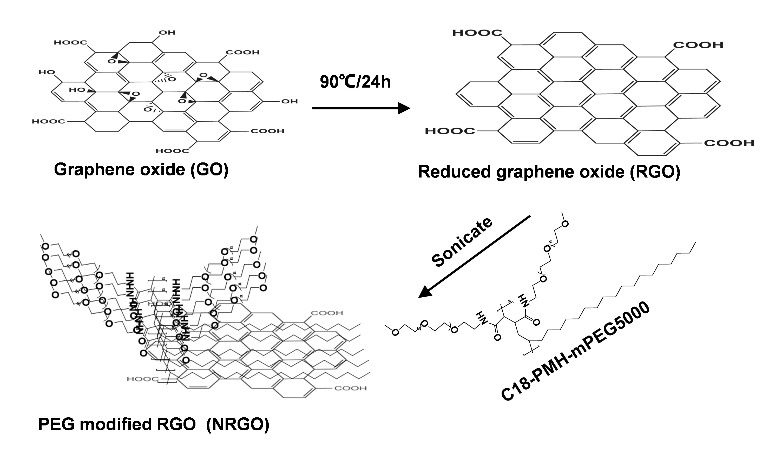


**Figure S1.** Preparation of the PEG modified reduced graphene oxide nanosheets (NRGO).

**Figure S2.** Temperature curves of PBS, NGO and NRGO under the irradiation with 808 nm laser (1W/cm^2^).


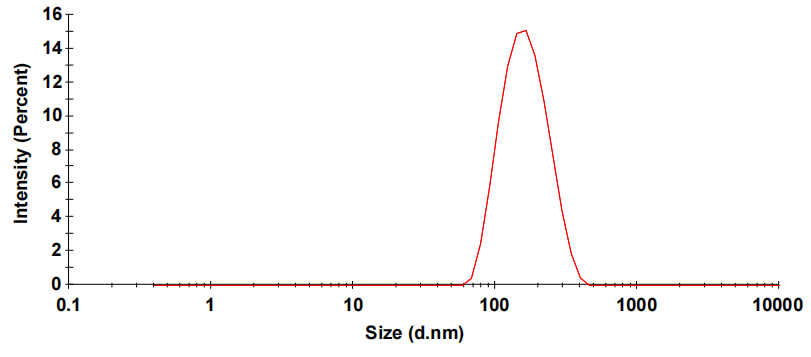


**Figure S3.** DLS analysis of particle size of S-G.

**Figure S4.** Cumulative release profiles of SFX from NRGO under different pH (6.0 and 7.4) with or without laser irradiation. Data are shown as means ± SEM (n = 3). The comparison of two groups was followed by unpaired Student’s t-test (two-tailed). *P < 0.05 and **P < 0.01.


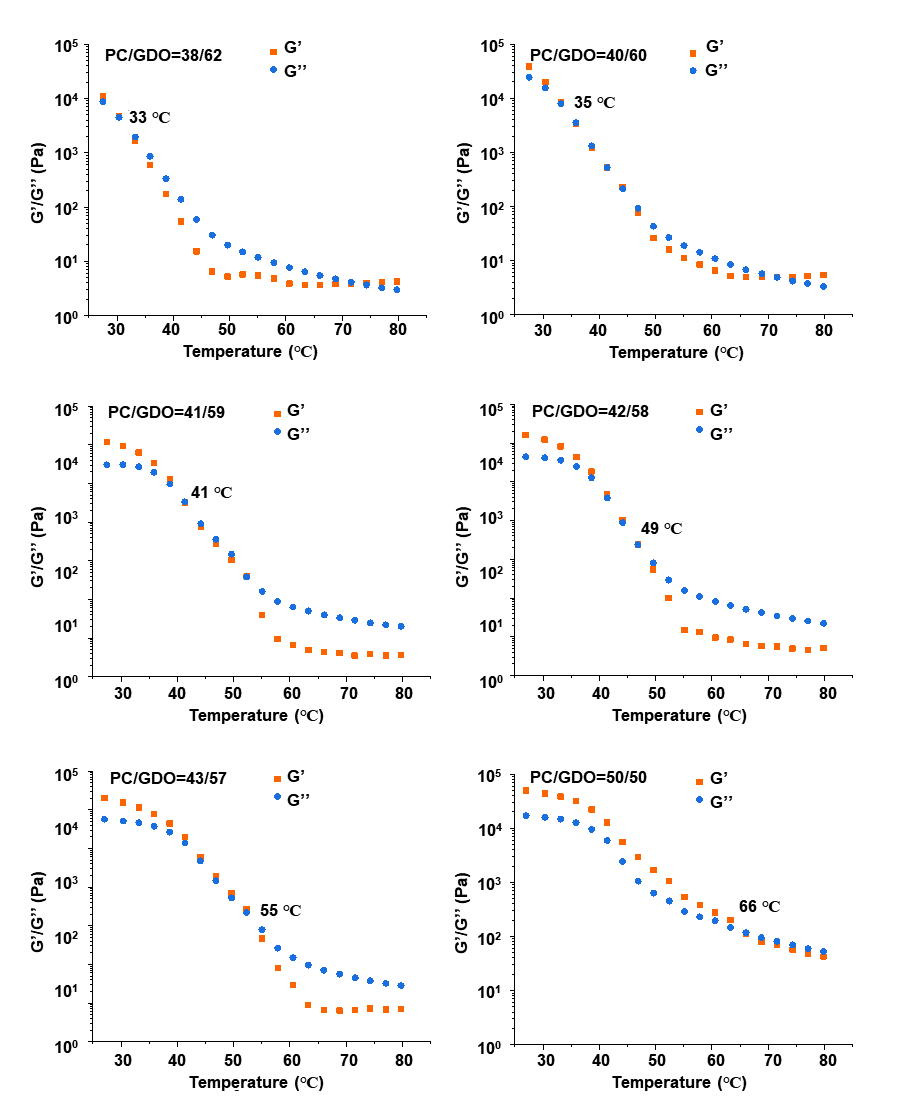


**Figure S5.** The phase transition temperature of LGs with different ratios of PC and GDO.


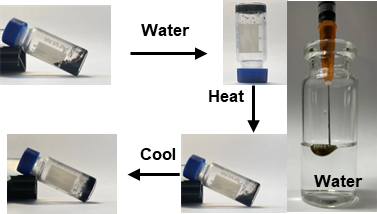


**Figure S6.** Photographs of the sol-to-gel transition upon adding water and changing the temperature.


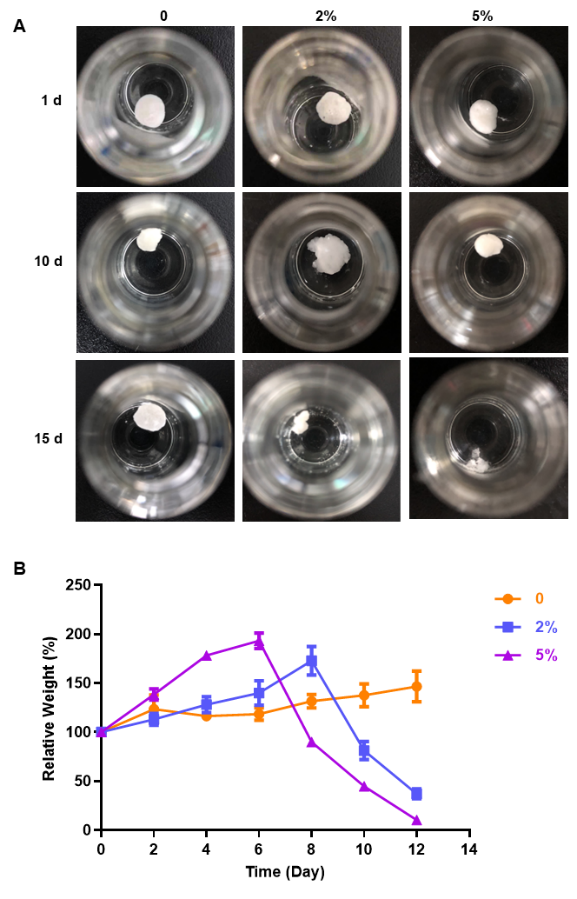


**Figure S7.** In vitro degradation behavior (A: morphology changes; B: degradation curves) of the gel incubated with 0%, 2%, and 5% of lipase over 14 d in PBS (pH 7.4), respectively.


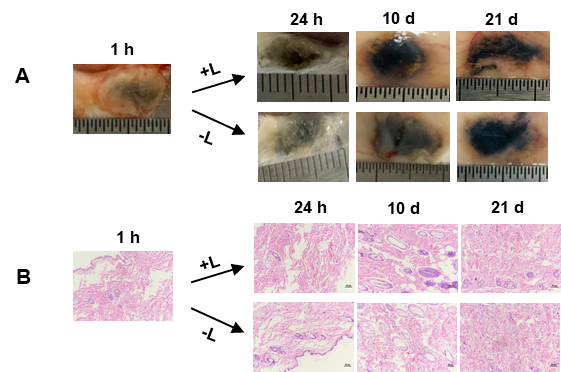


**Figure S8.** In vivo degradation behavior and tissue biocompatibility of the in situ-formed LG with H&E staining of the surrounding skin at different timepoints (scale bar: 50 μm).


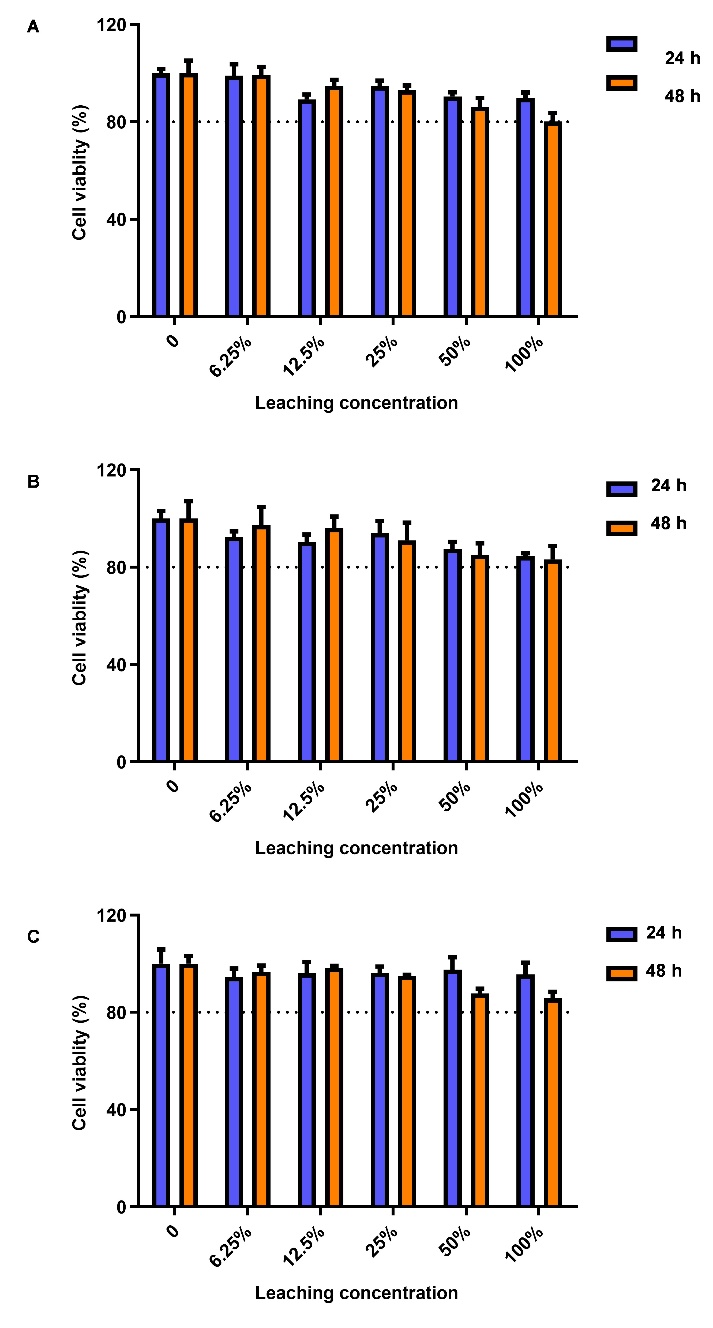


**Figure S9.** The cytotoxicity of LG against different cell lines at 24 h and 48 h. (n = 6). (A) 4T1, (B) HUVEC, (C) DCs.


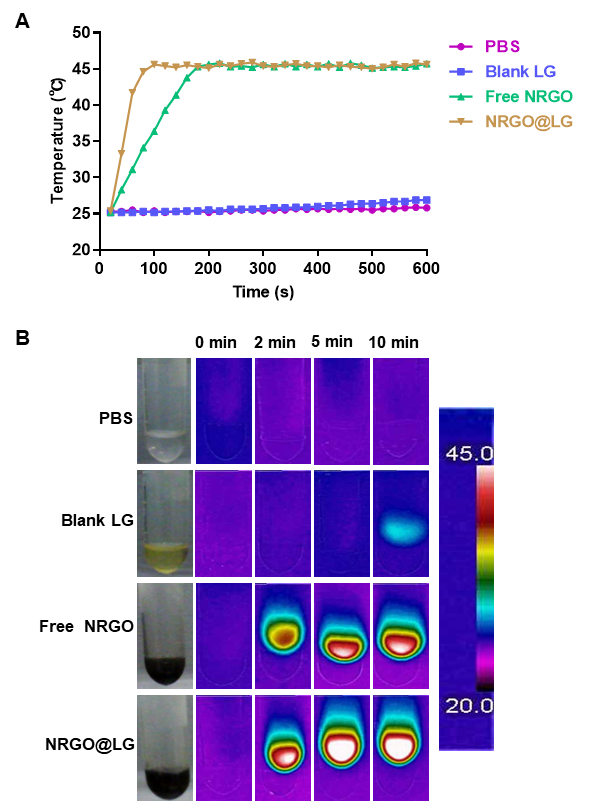


**Figure S10.** In vitro photothermal effect. (A) Temperature curves of PBS, blank LG, free NRGO and NRGO@LG with the NIR laser irradiation for 10 min in vitro. (B) In vitro infrared thermal images of PBS, blank LG, free NRGO and NRGO@LG before and 10 min after illumination.

**Figure S11.** Temperature curves of PBS, blank LG, free NRGO and NRGO@LG with the NIR laser irradiation for 10 min in vivo.


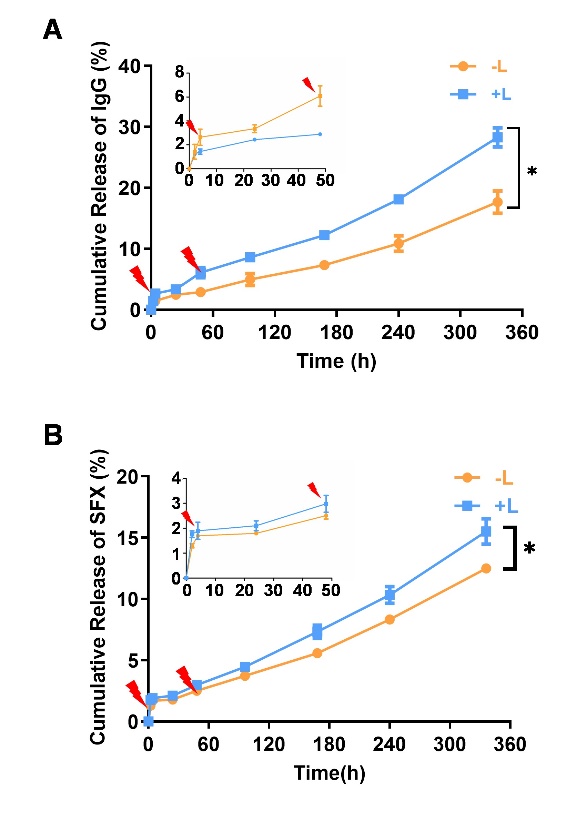


**Figure S12.** (A) Cumulative release profiles of IgG from IgG/S-G@LG incubated with PBS with or without 808 nm laser illumination. Data are shown as means ± SEM (n = 3). (B) Cumulative release profiles of SFX from IgG/S-G@LG incubated with PBS with or without 808 nm laser illumination. Data are shown as means ± SEM (n = 3). *P < 0.05.


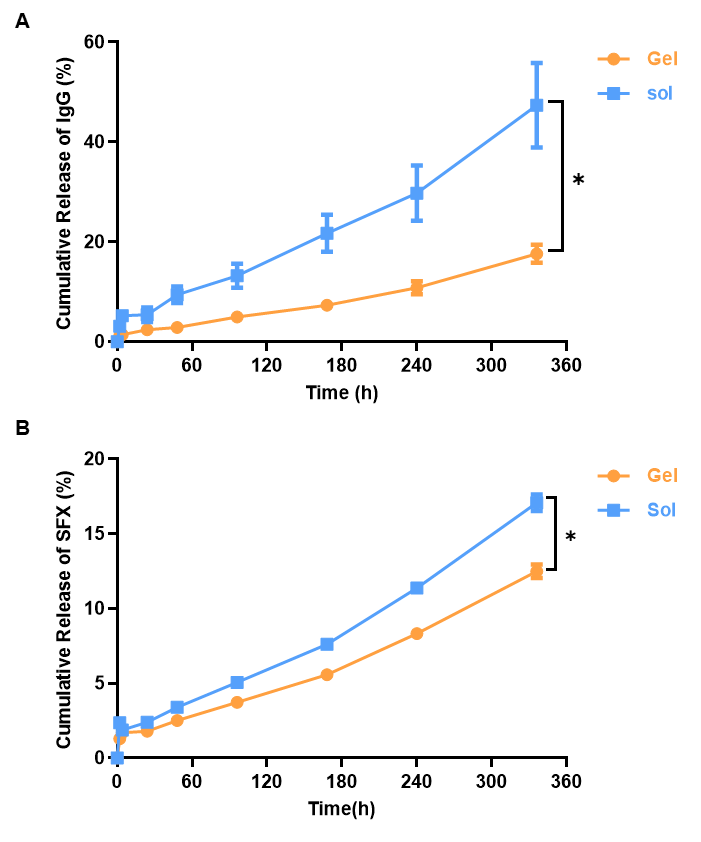


**Figure S13.** Cumulative release profiles of IgG (A) and SFX (B) from the sol or gel forms of LG in PBS. Data are shown as means ± SEM (n = 3). The comparison of two groups was followed by unpaired Student’s t-test (two-tailed). *P < 0.05.


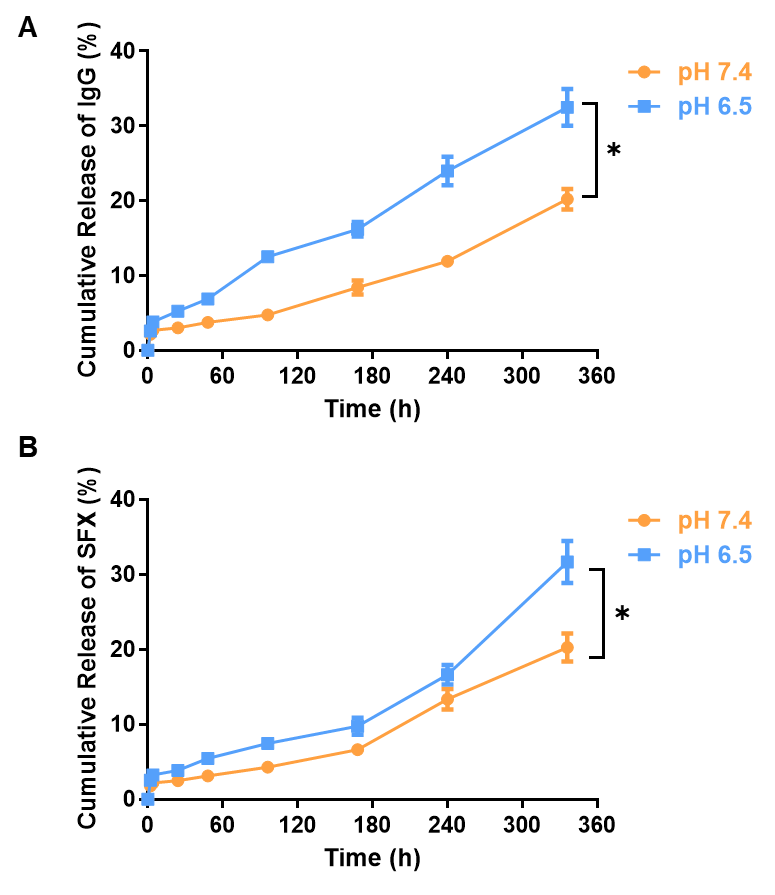


**Figure S14.** Cumulative release profiles of IgG (A) and SFX (B) of the LG in PBS under pH 6.5 and pH 7.4, respectively. Data are shown as means ± SEM (n = 3). The comparison of two groups was followed by unpaired Student’s t-test (two-tailed). *P < 0.05.


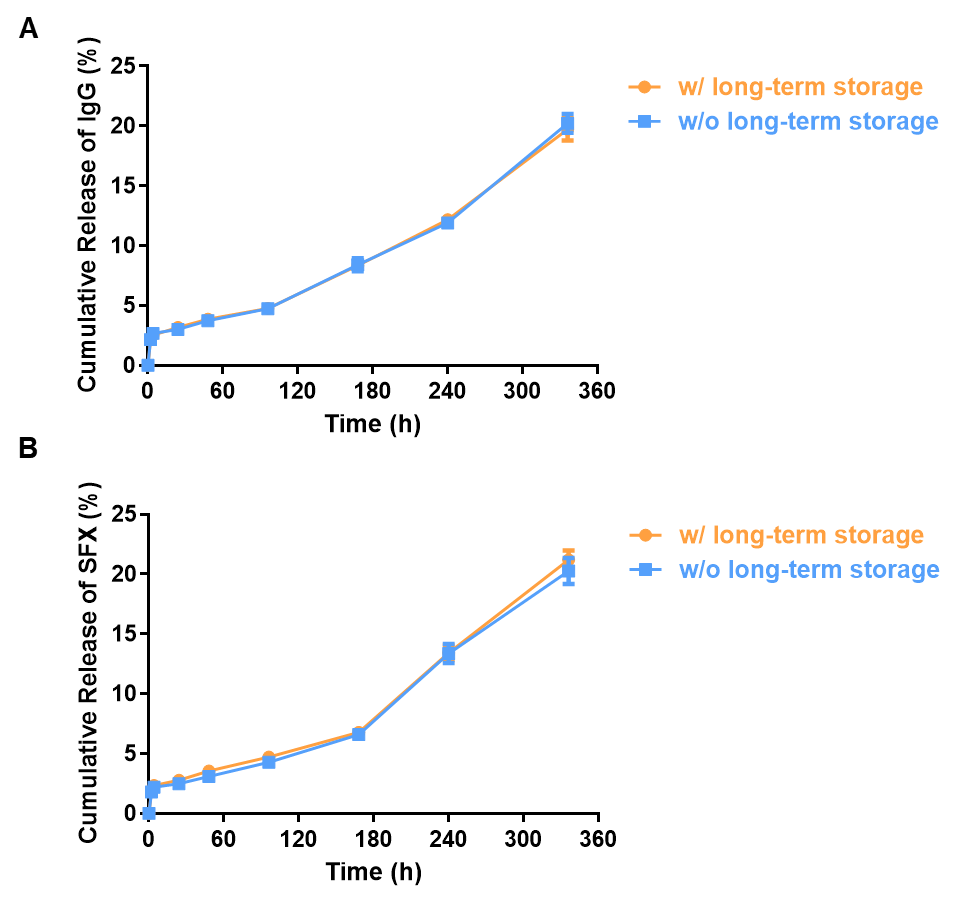


**Figure S15.** Cumulative release profiles of IgG (A) and SFX (B) of the LG with or without long-term storage, respectively. Data are shown as means ± SEM (n = 3). The comparison of two groups was followed by unpaired Student’s t-test (two-tailed). *P < 0.05.


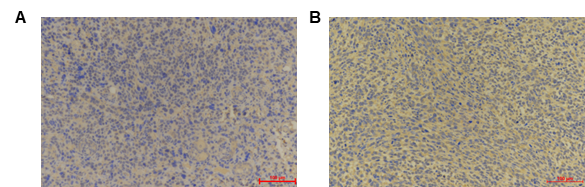


**Figure S16.** IHC plots of the expression level of PD-L1 in tumor tissues: (A) PBS; (B) mild PTT (45 ℃ for 10 min). Scale bar: 100 μm.

**Figure S17.** Size distribution of 4T1 cells derived-exosomes determined by NTA.


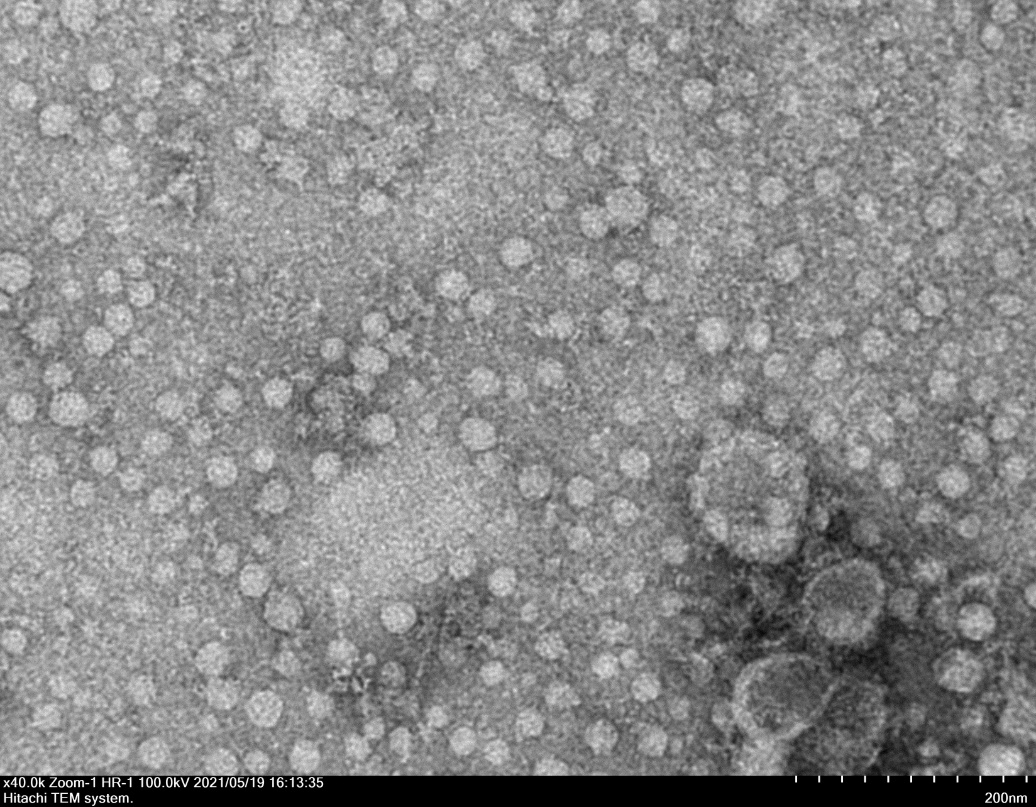


**Figure S18.** TEM images for 4T1 cells derived-exosomes.


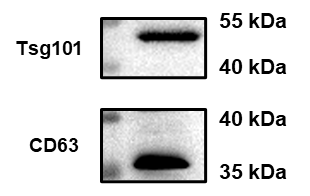


**Figure S19.** Western blot analysis of 4T1 cells derived-exosomes.

**Figure S20.** Quantification analysis of exosomes release after different treatments incubation with or without SFX (100 μM) under the temperature of 37 ℃ or 45 ℃ in vitro using 4T1-CD63-GFP cell lines.

**Figure S21.** Total exosomal protein per tumor weight after different treatments of MDA-MB-231 bearing mouse model. Data are shown as means ± SEM (n = 4). The comparison of two groups was followed by unpaired Student’s t-test (two-tailed). *P < 0.05 and ***P<0.001.

**Figure S22.** Relative PD-L1 expression of exosomes in TME after different treatments of MDA-MB-231 bearing mouse model. Data are shown as means ± SEM (n = 4). The comparison of two groups was followed by unpaired Student’s t-test (two-tailed). *P < 0.05.

**Figure S23.** The body weight change curve of the 4T1-bearing mouse models. Data are shown as means ± SEM (n = 5).


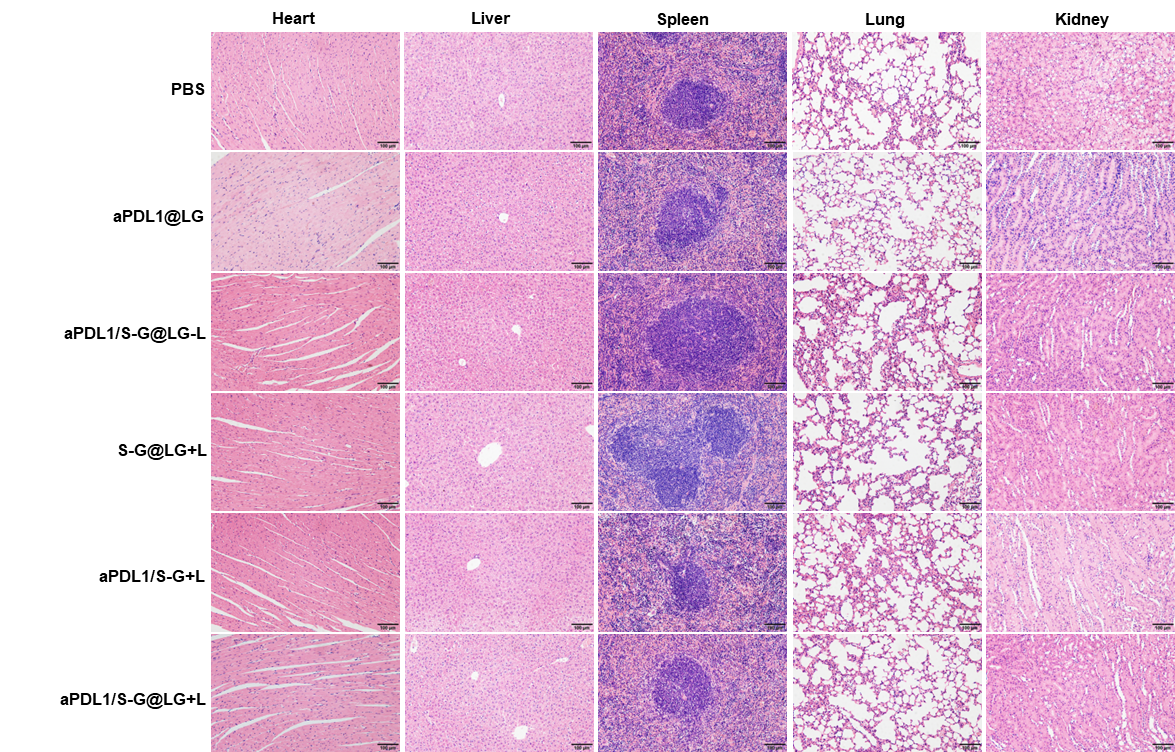


**Figure S24.** Histopathological images of the main organs, containing liver, lung, kidney and heart obtained from the 4T1-bearing mice.

**Figure S25.** Absolute numbers of CD4^+^ T cells in the primary tumors after different treatments examined on day 8 after treatment. Data are shown as means ± SEM (n=3). The comparison of two groups was followed by unpaired Student’s t-test (two-tailed). *P<0.05 and **P<0.01.


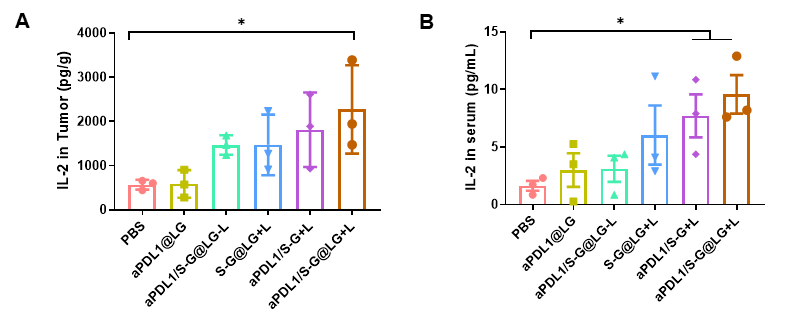


**Figure S26.** Cytokine levels of IL-2 in the tumor tissues (A) and serum (B) from mice isolated 8 days after different treatments. Data are shown as means ± SEM (n=3). The comparison of two groups was followed by unpaired Student’s t-test (two-tailed). *P<0.05.


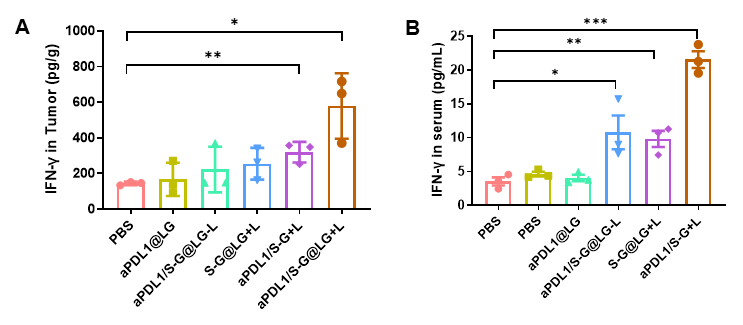


**Figure S27.** Cytokine levels of IFN-γ in the tumor tissues (A) and serum (B) from mice isolated 8 days after different treatments. Data are shown as means ± SEM (n=3). The comparison of two groups was followed by unpaired Student’s t-test (two-tailed). *P<0.05, **P<0.01 and ***P<0.001.


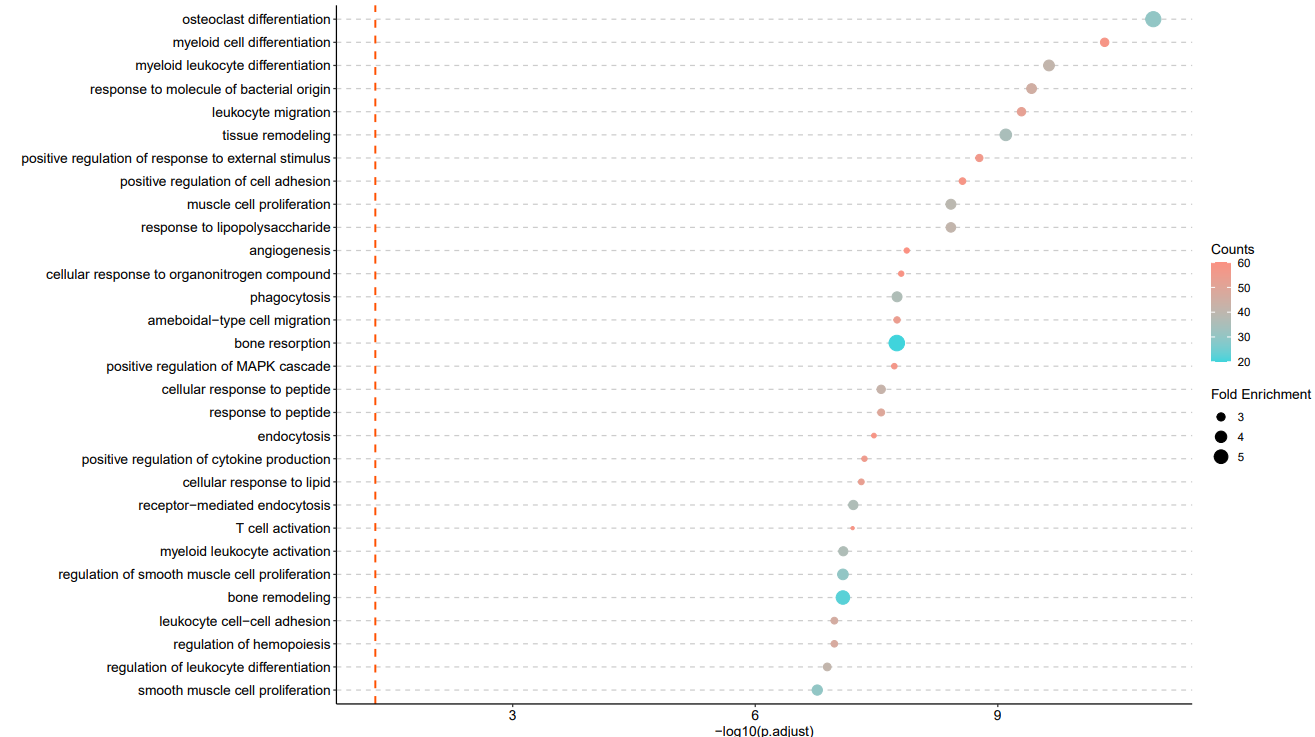


**Figure S28.** KEGG enrichment analysis of the TILs between S-G@LG+L and NRGO@LG+L treated tumors (analysis was performed by one-sided hypergeometric distribution test followed by false discovery rate correction).

**Figure S29.** Absolute numbers of CD45^+^ lymphocytes in the distal tumors after different treatments examined on day 8 after treatment. Data are shown as means ± SEM (n=3). The comparison of two groups was followed by unpaired Student’s t-test (two-tailed). *P<0.05.


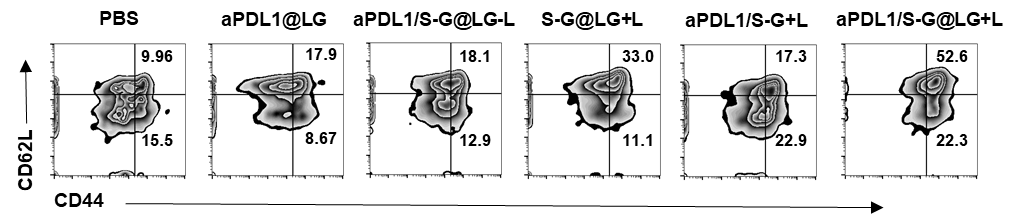


**Figure S30.** Representative flow cytometry plots showing different groups of T_EM_ and T_CM_ in LNs (gated on CD3^+^CD8^+^ T cells).
